# A systematic review of DoS and DDoS attack detection in cloud environments: evolutionary trends and the emerging role of meta-learning for cross-domain adaptability

**DOI:** 10.3389/frai.2026.1958096

**Published:** 2026-10-21

**Authors:** Amit Tiwari, C. Navaneethan

**Affiliations:** 1 School of Computer Science Engineering and Information System, Vellore Institute of Technology, Vellore, Tamil Nadu, India; 2 Department of Software and Systems Engineering, School of Computer Science Engineering and Information System, Vellore Institute of Technology, Vellore, Tamil Nadu, India

**Keywords:** deep learning, distributed denial of service, Internet of Things, intrusion detection systems, machine learning, meta-learning, software-defined networking, transfer learning

## Abstract

Denial of Service (DoS) and Distributed Denial of Service (DDoS) attacks continue to be one of the most persistent and rapidly evolving threats to current network infrastructures as they are becoming more sophisticated and increasingly disrupting the availability, reliability and security of cloud-based systems, Software-Defined Networks (SDNs), Internet of Things (IoT) ecosystems and next generation communication environments. This paper provides a systematic review of the literature on DoS and DDoS attack detection and mitigation, covering the various stages of evolutions in the field from early statistical and anomaly-based methods to adaptive machine learning and deep learning, as well as domain specific detection methods. The review, which is based on a thorough examination of 120 papers (114 primary studies and 6 supplementary studies), provides a synthesis of the latest advances in network and application-layer detection, protocol-specific detection frameworks, cloud-based intrusion detection systems, SDN-enabled programmable detection, IoT/Fog/Edge architectures, ensemble and hybrid models, transfer learning, and blockchain-based security mechanisms. A systematic review of benchmark datasets, feature engineering techniques and comparative performance shows that current deep learning systems attain almost perfect classification rates in experimental environments, but fundamental limitations in their generalizability, computational efficiency, preservation of privacy and explainability remain a constraint for their deployment in real-world environments. Importantly, this is a lot of work, but less than 6 % of the reviewed studies (seven out of 120) use meta-learning, transfer learning or few-shot approaches explicitly, which reflects the ongoing lack of capability in the field of adaptive generalization and a deficiency in addressing zero-day attacks and domain shift in dynamic cloud environments. We propose the emerging paradigm of meta-learning as the most promising one for next-generation cloud-based intrusion detection, which can be adapted to new domains with very few labeled examples — directly overcoming the three key limitations of existing approaches, namely the zero-day detection gap, domain shift limitations and label scarcity. This review offers a structured basis for researchers and practitioners designing explainable, privacy-preserving and computationally efficient detection systems and highlights the critical need for moving along the path of designing cloud-based intrusion detection systems based on meta-learning.

## 1 Introduction

The widespread development of digital infrastructure within cloud computing environments, software-defined networks, and Internet of Things (IoT) systems has radically altered the new computing paradigm, which has also increased the threat spectrum that organizations face today around the world (Osanaiye et al., 2016; Singh et al., 2016). Denial of Service (DoS) and Distributed Denial of Service (DDoS) attacks remain some of the most widespread and disruptive types of cyber-attacks that still present unprecedented challenges to service and infrastructure integrity and economic sustainability (Osanaiye et al., 2016; Somani et al., 2016). These attacks work on the principle of saturating target systems with malicious traffic to deplete computational or network resources, and have changed significantly in magnitude, complexity, and variety in the last 10 years (Gulisano et al., 2015; Wang et al., 2015). Volumetric flooding attacks, and covert low-rate application-layer intrusions are only a few examples of a more complex adversarial landscape that requires corresponding improvements in detection and mitigation technologies (Mitchell and Chen, 2014; Liang and Znati, 2019).

DoS and DDoS attacks are of significant global effect and have been well documented in the research literature. As Osanaiye et al. (2016) emphasized, cloud computing environments present unique architectural weaknesses that make traditional DDoS defences too weak and instead require adaptive and distributed mitigation frameworks tailored to deploying cloud native. Empirical analysis by Somani et al. (2016) revealed that DDoS attacks in multi-tenant cloud infrastructure cause distressing collateral damages to non-target co-resident tenants, leading to cascading resource overconsumption and over migration of virtual machines that worsen the quality of the overall service delivery. Singh et al. (2016) also emphasized that the security of cloud computing is multi-layered as it involves threats on an infrastructure, platform and application service layers which cannot be mitigated through a single protective mechanism. All these findings jointly prove that the cloud environment is not a passive hosting medium, but rather an active and dynamic attack surface that needs smart, contextual and constantly adaptive protection systems (Singh et al., 2016; Coppolino et al., 2017).

These properties of cloud computing that render it appealing, such as provisioning of resources on-demand, multi-tenancy, virtualization, and on-demand scalability, also present peculiar security challenges that differentiate cloud-based environments with a traditional network infrastructure (Singh et al., 2016; Iqbal et al., 2016). Resource sharing across multiple tenants in clouds opens the possibility of co-resident attacks, side-channel attacks, and Economic Denial of Sustainability attacks that disrupt the financial model of cloud service consumption and not only its availability (Somani et al., 2016; Shah et al., 2021). The interplay between cloud computing and Software-Defined Networking, edge computing, and IoT also widens the attack surface even further, as the nature of traffic becomes highly dynamic, and patterns of attacks become even harder to differentiate between normal workload variations and malicious intent (Wang et al., 2015; Sharma et al., 2018). Wang et al. (2015) have shown that even advanced probabilistic detection models can be affected under dataset shift in SDN-integrated cloud settings, necessitating periodic model validation to retain detection performance-a factual drawback that prompts the creation of naturally adaptive detection models.

Conventional Intrusion Detection Systems which are mostly based on signature and rule-based approaches have continued to become inadequate against the changing attack patterns and the dynamic nature of traffic in the contemporary cloud environments (Mitchell and Chen, 2014; Lata and Singh, 2022). In their extensive review of wireless network IDS methods, Mitchell and Chen (2014) reached the conclusion that no single method of detection can be universally the best in a wide variety of deployment scenarios, and that the lack of common evaluation metrics essentially precludes any sensible comparative analysis and useful advice to deployment. Casas et al. (2012) showed that sub-space clustering and evidence accumulation can be used to detect unknown attacks on more typical benchmark datasets but admitted the inherent difficulty in dynamic models that rely on historical traffic distributions to be able to effectively adapt to unseen attack behavior. This point was further solidified by Lata and Singh (2022) in their literature review of cloud IDS systematized the identification of high false alarm rates, limitations to scalability and failure to detect zero-day attacks in cloud-based intrusion detection systems, as a persistent and unresolved issue. Iqbal et al. (2016) also noted that to succeed in mitigating the various and dynamic attack vectors that target cloud infrastructures, intrusion detection and prevention systems need to cut across all layers of the cloud service, which is essentially an impossible requirement of the simple rule-based systems.

The shortcomings of the methods of detection that are based on the static and threshold are also demonstrated throughout a collection of contributions in the literature. As Jing et al. (2019) noted, sketch-based traffic fusion algorithm in the detection of DDoS is extremely sensitive to tuning of parameters and less reliable when used in different traffic settings. Similar weaknesses in dynamic thresholding schemes were reported by David and Thomas (2019), which exhibit impaired performance to stealthy traffic or steeply varying traffic patterns. Liang and Znati (2019) proved that no individual machine learning method is always more efficient than others in various types of traffic, different sets of features, and attack severity, especially when the class imbalance conditions typical of real-world data are considered. Prasad et al. (2020a) also observed that hybrid classifiers demonstrate lower performance in situations with highly imbalanced and rare attack classes which highlights the sensitivity of trained models to dataset composition. All these deficiencies underscore the critical importance of detection frameworks that are self-adaptive to non-stationary traffic distributions, and do not need to retrain entirely (Liang and Znati, 2019; Lata and Singh, 2022).

Machine Learning and Deep Learning have introduced revolutionary possibilities to overcome the shortcomings of traditional IDS in cloud and network security scenarios (Kanimozhi and Prem Jacob, 2019; Garg et al., 2020). (Kumar and Selvakumar, 2013) introduced an ensemble Neuro-Fuzzy classifier, which is a combination of Adaptive Neuro-Fuzzy Inference Systems and boosting techniques, which show lower false positive rates than traditional ensemble classifiers in several benchmarks datasets. The study by Kanimozhi and Prem Jacob (2019) showed that hyper-parameter-optimization of Artificial Neural Networks that have been trained using realistic cloud intrusion data yields very robust detection of botnet and contemporary intrusion. Garg et al. (2020) suggested an ensemble Artificial Bee Colony-based anomaly detection model that combines Restricted Boltzmann Machines and adaptive clustering, which showed that the hybrid model performs better than the traditional supervised and unsupervised models on a variety of cloud datasets. Hosseini and Azizi (2019), additionally revealed that hybrid detection based on a Random Forest possesses strong DDoS classification on both standard benchmark and custom datasets.

Deep learning architectures have been demonstrated to be superior to shallow models in terms of temporal and sequence modeling of network traffic, with complex dependencies that shallow models cannot model (Priyadarshini and Barik, 2022; Deniz and Serttaş, 2023; Issa and Albayrak, 2023). A model based on the LSTM was suggested by Priyadarshini and Barik (2022) in the framework of an SDN-enabled fog-cloud architecture and showed effective DDoS mitigation with high cross-validation rates. Issa and Albayrak (2023) suggested a hybrid CNN-LSTM model and showed that the performance of intrusion detection in this model is better than that of either of the two architectures. A balanced random sampling-based CNN framework to detect real-time DDoS in SDN environments was presented by Najar and Naik (2024), which showed up to almost perfect binary and multi-class detection. Shaikh et al. (2024) also established that integrating Vision Transformers with CNN models enhances the ability of DDoS detection as compared to the performance of standalone convolutional models.

Nevertheless, even with these developments, there are still critical constraints which drive further studies on adaptive and generalizable detection processes (Abdullayeva, 2023; Abdallah et al., 2024; Afraji et al., 2025). Abdullayeva (2023) noted that deep learning models need a high quality and variety of datasets to have generalization, and Afraji et al. (2025) found that the unavailability of extensive datasets specific to clouds is a critical bottleneck to deep learning-based defence strategies. Mandela and Etyang (2025) emphasized that deep learning model has always struggled to keep up with changing attack patterns because the model is trained in a static framework. Abdallah et al. (2024) have also found that even the most advanced systems still face the problem of scalability, heterogeneity of the data, and privacy limitations, and Lata and Singh (2022) have also supported the necessity to have fundamentally adaptive learning paradigms in the future of intrusion detection.

All these limitations are indicative of meta-learning as an untapped and promising paradigm in cybersecurity detection (Farzaneh et al., 2024; Abdallah et al., 2024). Though Farzaneh et al. (2024) have proved that deep transfer learning is very useful in the context of limited labeled data to detect DDoS, the systematic deployment of meta-learning to adaptive DoS/DDoS detection in cloud environments has not been critically investigated. The available reviews by Ali et al. (2023b) and Abdallah et al. (2024) are useful in terms of the comparison of ML and DL, however, they do not analyze the fast-adaptation and few-shot learning property of meta-learning in cloud-based intrusion detection when the data distribution is shifted.

It is against this background that the present systematic review has been conducted. Existing surveys have been useful in other areas of cloud intrusion detection, such as Lata and Singh (2022) who reviewed and summarized cloud IDS architectures and highlighted problems of scalability and false alarms, and Abdallah et al. (2024) who comparatively compared ML and DL-based anomaly detection methods for cloud network security, while neither asked the specific question of meta-learning as a cloud IDS detection paradigm nor looked at problems of fast adaptation, few-shot generalization or cross-domain transferability in the context of detecting DoS and DDoS attacks. To the best of the authors’ knowledge, there is no prior systematic review that addresses the intersection of meta-learning, adaptive detection mechanisms and DoS/DDoS attack detection in cloud computing environments, which is the focus of this review. The existing literature remains divided between technique-specific assessments and domain-specific surveys, neither of which adequately addresses the adaptive demands of the cloud-native threat landscape (Lata and Singh, 2022; Afraji et al., 2025). This paper presents a systematic review of 120 research papers spanning DoS/DDoS detection, cloud security, ML and DL-based classification, transfer learning, and adaptive cybersecurity frameworks (Kumar and Selvakumar, 2013; Garg et al., 2020; Najar and Naik, 2024; Shaikh et al., 2024). There are a few survey and review papers that have discussed some facets of cloud security, DDoS detection, and intrusion detection using ML, but there is no single paper that provides a thorough treatment on all of these aspects in an integrated framework that incorporates meta-learning, adaptive detection and systematic PRISMA-compliant approach. To situate the present review in the context of the other twelve survey and review papers that make up the corpus, the scope, limitations and key findings of each are summarized in Table 1.

**Table 1:** Summary of survey and review papers in the corpus.

Survey	Scope	Limitation	Key Finding
Mitchell and Chen (2014)	Wireless network IDS across WLAN, WSN, *ad hoc*, and CPS environments	Lacks unified evaluation metrics and comprehensive comparative analysis	No single detection approach is optimal for all wireless network environments
Osanaiye et al. (2016)	DDoS attacks and mitigation strategies in cloud computing environments	Conceptual framework without experimental validation using real-world datasets	Cloud-specific characteristics require adaptive and distributed mitigation frameworks
Singh et al. (2016)	Security issues, threats, and mitigation strategies in cloud computing	Conceptual analysis without experimental validation or quantitative evaluation	Cloud security requires multi-layered protection across infrastructure, platform, and application layers
Iqbal et al. (2016)	Security attack taxonomy and IDPS across SaaS, PaaS, and IaaS cloud layers	Proposed taxonomy and IDS framework lack experimental validation	Cloud Security requires integrated IDPS service across all cloud service layers
Coppolino et al. (2017)	Cloud security threats and solutions across network, hypervisor, and hardware	Many proposed mechanisms are immature and not suitable for operational deployment	Multi-layered security combining network, hypervisor, and hardware defences is required
Khoda Parast et al. (2022)	Security of IaaS, PaaS, and SaaS service models including DoS/DDoS threats	PaaS security vulnerabilities and countermeasures are insufficiently explored	DoS/DDoS attacks and shared virtualization technologies are the most critical cloud threats
Lata and Singh (2022)	Cloud IDS across IaaS, PaaS, and SaaS including network-based and host-based IDS	High false alarm rates, scalability issues, and inability to detect zero-day attacks	Hybrid IDS with feature selection and optimized deployment is essential for effective cloud intrusion detection
Abdullayeva (2023)	Cyber resilience and security reference model for intelligent cloud systems	Theoretical framework without experimental evaluation on real attack datasets	Comprehensive cyber security reference model must address all cloud layers including virtualization and IoT
Ali et al. (2023b)	ML and DL techniques for DDoS detection in SDN environments	Does not implement or evaluate models experimentally on a unified dataset	Deep learning outperforms traditional ML for DDoS detection in SDN environments
Asha and Shanmugapriya (2024)	Insider threat taxonomy, incident analysis, and detection in cloud organizations	Lack of real-world insider threat datasets and over-reliance on synthetic data	Insider threat requires integrated taxonomy-driven detection due to trusted access and data imbalance
Abdallah et al. (2024)	Cloud network anomaly detection using ML and DL techniques	Limited to literature review without experimental validation on a unified dataset	ML and DL improve cloud anomaly detection, but scalability, heterogeneity, and privacy remain challenges
Afraji et al. (2025)	DL-based defence strategies for DDoS mitigation in cloud and IoT environments	Constrained by lack of comprehensive cloud-specific DDoS datasets	DL enhances DDoS mitigation in cloud but requires better datasets and explainable AI integration
Proposed Review	DoS/DDoS detection across cloud, SDN, IoT, and edge-120 studies 2012–2026	Single-reviewer screening; no protocol registration	Meta-learning is identified as the critical missing paradigm for adaptive zero-day DoS/DDoS detection in cloud environments

As can be seen in Table 1, previous surveys focus on single topics of cloud security, ML-based cloud detection, or DDoS mitigation, but neither survey provide a detailed focus on either meta-learning or few-shot generalization, or on adaptive detection paradigms.

The contributions of this review are four-fold: (1) a systematic taxonomic analysis tracing the evolutionary trends in detection methods from 2012–2026 (Kumar and Selvakumar, 2013; Issa and Albayrak, 2023; Shaikh et al., 2024); (2) a critical assessment of datasets, performance metrics, and methodological shortcomings across the reviewed literature (Liang and Znati, 2019; Lata and Singh, 2022; Abdallah et al., 2024); (3) a synthesis of adaptive and transfer learning models that identifies and formally defines the meta-learning research gap in cloud-based intrusion detection (Farzaneh et al., 2024; Afraji et al., 2025); and (4) a set of actionable directions for developing meta-learning-driven, cross-domain-adaptive detection frameworks capable of addressing zero-day attacks and domain shift in real-world cloud deployments.

The rest of this paper is structured in the following way. Section 2 gives the methodology and selection criteria of the review. Section 3 is the complete literature review of DoS and DDoS detection techniques over statistical, machine learning, deep learning, and domain-specific models. Section 4 discusses feature engineering methods used in the studies reviewed. Section 5 discusses the evaluation metric used in the studies reviewed. Section 6 explains benchmark datasets that have been used in the literature and their features and adoption trends. Section 7 presents a comparative discussion of the current detection methods. Section 8 determines the main research issues and gaps in the area. Section 9 provides future research directions and Section 10 provides a conclusion of the paper.

## 2 Review methodology

### 2.1 Overview of the review protocol

The Preferred Reporting Items for Systematic Reviews and Meta-Analyses PRISMA 2020 guidelines (Page et al., 2021) were followed in developing this systematic review, which offers a structured and transparent approach to systematically identify, screen, evaluate, and synthesize relevant research literature. A PRISMA 2020 checklist is included with this submission as a supplementary document, for each of the 27 items the location in the manuscript is indicated. This review has not been registered in a prospective register before it was begun. Failure to register their work in advance is recognized as a methodological limitation and is in line with the type of work being done, which is a domain specific systematic literature review in computer science instead of a clinical or epidemiological meta-analysis. The main goal of the review protocol was to guarantee that the research landscape in the field of DoS and DDoS attack detection in cloud-based systems is fully covered, repeatable and bias-free, paying special attention to adaptive learning mechanisms and meta-learning-based techniques. The protocol was intended to reduce selection bias, be reproducible and generate a corpus of papers that covers the entire temporal and thematic depth of the domain between 2012 and 2026. Methodological quality was considered informally during synthesis to guide the depth of discussion assigned to individual studies, but no formal scoring instrument was applied. The review process is documented in the PRISMA 2020 flow diagram provided as Figure 1 and the completed PRISMA checklist provided as Supplementary material.

**Figure 1:**
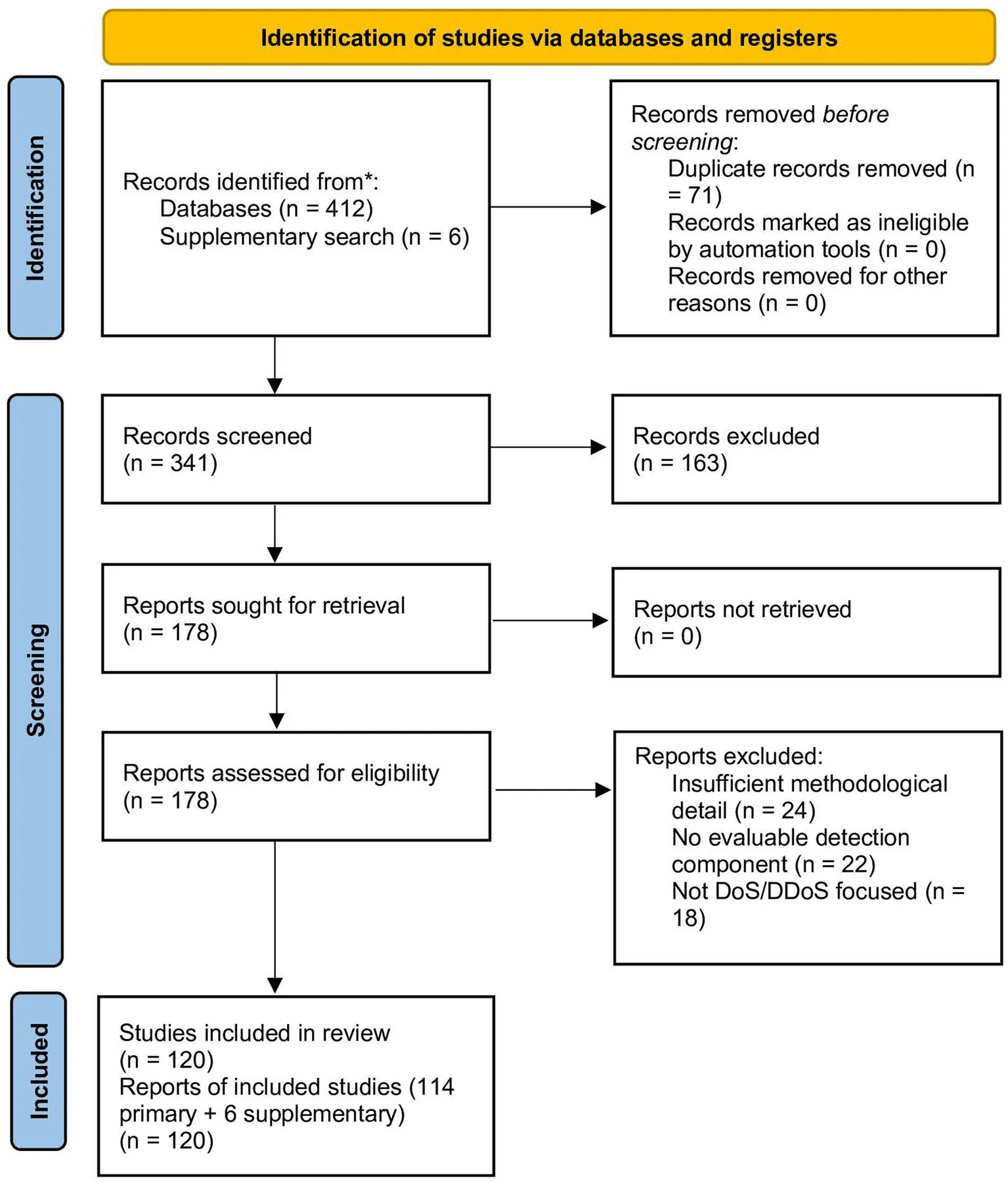
PRISMA 2020 flow diagram illustrating the systematic paper selection and screening process for the review of DoS and DDoS attack detection studies. Source: Page et al. (2021).

### 2.2 Research question

The review was structured into four research questions, around which the paper selection, classification and synthesis were done:

*RQ1*: What conventional machine learning and deep learning approaches have been used to detect DoS and DDoS attacks in network and cloud computing environment from 2012 till 2025?

*RQ2*: What are the typical datasets and metrics used and which are the typical methodologies employed in AI-based intrusion studies and what are the similarities and differences of these studies?

*RQ3*: What are the main problems and restrictions of the existing detection techniques based on ML and DL in terms of adaptability, generalization and real world deployment in cloud infrastructure?

*RQ4*: What are the research works on adaptive learning paradigms (especially transfer learning and meta-learning) for DoS/DDoS detection so far and what are the gaps in the research?

The Table 2 represents the PICOS framework for this systematic review.

**Table 2:** PICOS framework for this systematic review.

Element	Definition for this review
P- Population	Network and cloud computing environments subject to DoS and DDoS attacks
I- Intervention	AI, machine learning, deep learning, and adaptive detection mechanisms
C- Comparator	Traditional signature-based and threshold-based IDS approaches
O- Outcome	Detection accuracy, false positive rate, false negative rate, detection latency, and generalization to novel attacks
S- Study Design	Peer-reviewed journal articles and conference papers with experimental evaluation

All of these questions guaranteed that the review covered not only the state of the art in technical aspects, but also the methodological and deployment-oriented gaps to be addressed by the development of meta-learning-based adaptive detection framework. Table 3 maps each research question to the section or sections of this review that directly address it.

**Table 3:** Representation of mapping of research question to the concerned sections.

Research question	Addressed in
RQ1	Section 4, 5
RQ2	Section 6, 7
RQ3	Section 7, 8
RQ4	Section 6.5, 8,9

### 2.3 Literature search strategy

A systematic search was done on five major academic databases: IEEE Xplore, ScienceDirect (Elsevier), SpringerLink, ACM Digital Library and Google Scholar. The databases were chosen because of the fact that they include a comprehensive coverage of high impact computer science and cybersecurity publications which includes both journals and conference proceedings. The main search took place from January 2025 until March 2025. Only publications between January 2012 and March 2025 were searched.

The search strategy used involved searching using Boolean combination of the keywords grouped in three conceptual clusters. The first cluster was for the attack domain and contained the following terms: DoS attack,” “DDoS attack,” “distributed denial of service,” “denial of service,” “flooding attack”, and “intrusion detection system. The second cluster was focused on the deployment environment, which comprised the topics of “cloud computing,” “cloud security,” “software-defined networking,” “fog computing,” “edge computing,” and “IoT security. The third cluster had to do with the learning paradigm, where “machine learning,” “deep learning,” “neural network,” “transfer learning,” “ensemble learning,” “anomaly detection,” and “adaptive detection” were the topics covered. The following Boolean string was used as the main query in all five databases:

(“DoS attack” OR “DDoS attack” OR “distributed denial of service” OR “denial of service” OR “flooding attack” OR “intrusion detection” OR “cloud computing” OR “cloud security” OR “software-defined networking” OR “fog computing” OR “edge computing”) AND (“IoT security” OR “machine learning” OR “deep learning” OR “neural network” OR “anomaly detection” OR “ensemble learning”)

A supplementary search was then carried out to specifically detect few-shot learning and meta-learning literature that does not appear in the primary search terms, attack domain and cloud environment. The following Boolean string was used to search the five databases as a supplement:

The following keywords were used for title and abstract: (“meta-learning” OR “few-shot learning” OR “MAML” OR “prototypical network” OR “transfer learning” OR “domain adaptation”) AND (“intrusion detection” OR “DDoS” OR “DoS” OR “network security” OR “cloud security”).

The supplementary search further revealed six additional papers to the main search strings (Bellmunt et al., 2025; Chen and Ma, 2026), which were not picked up by the primary search strings, thus substantiating that the original corpus contained too few papers from the meta-learning field. These papers have been incorporated into the revised corpus, bringing the total to 120 studies.

### 2.4 Inclusion and exclusion criteria

An organized list of inclusion and exclusion criteria was formulated before the screening stage so that there are consistency and objectivity in the selection of papers and these are summarized in Table 4.

**Table 4:** Selection of papers based on inclusion and exclusion criteria.

Criterion	Inclusion	Exclusion
Publication type	Journal articles, conference papers, systematic reviews	Book chapters, theses, grey literature, editorials
Publication period	2012–2026	Before 2012 or after 2026
Language	English only	Non-English publications
Domain focus	DoS/DDoS detection, cloud security, IDS, network intrusion	Unrelated cybersecurity domains
Technical focus	ML, DL, statistical, or adaptive methods for attack detection	Paper with no detection component
Environment	Cloud, network, SDN, IoT, fog, edge environments	Purely physical or hardware-only security without software detection
Availability	Full-text accessible	Abstract-only or inaccessible papers

Articles were included when they suggested, applied, or tested a detection, mitigation or classification system against DoS or DDoS attacks using any type of AI, machine learning, deep learning, statistical learning, or adaptive system. Included in the survey and review papers were those papers that offer a systematic comparative analysis of detection techniques in the context of the research questions. Articles were filtered out based on not being involved with cybersecurity issues beyond the scope of DoS and DDoS detection, not including an experimental assessment or empirical validation or offering theory-based security models but without detectable detection elements.

### 2.5 Paper selection and screening process

The paper selection process was executed in four sequential stages. All screening and selection decisions were made by a single reviewer- the first author- under the guidance and final validation of the supervising author. Independent duplicate screening by a second reviewer was not performed, which is acknowledged as a limitation of this review. Consistency across screening decisions was maintained through the strict application of the pre-defined inclusion and exclusion criteria. The absence of dual independent screening and the consequent inability to report an inter-rater agreement statistic such as Cohen’s kappa are reported as limitation in Section 8. The initial search of the databases with the help of the selected keywords produced in the first stage a total of 412 promising papers in all five databases. In the second step, duplicate records were found and eliminated, and the candidate pool was narrowed to 341 unique papers. In the third step, titles and abstracts were filtered based on the inclusion and exclusion criteria, and 163 articles were excluded due to being either off-topic, not within the period or not available in full-text format and 178 articles were included in the full-text review. In the fourth step, full-text screening was done against all inclusion criteria where 64 other papers were further eliminated because of lack of methodological specificity, no evaluable experimental outcomes or because of not discussing DoS or DDoS detection in an AI-driven framework. This process yielded a final corpus of 120 papers that form the basis of this review.

### 2.6 Classification framework

The 120 selected papers were categorized based on a multi-dimensional taxonomy that was created in this review, summarized in Table 5. The taxonomy classifies papers on four dimensions: year of publication, main method of detection, the environment in which it is deployed, and the topic. This categorization allowed systematic cross-dimensional analysis and helped to identify the trends in time, gaps in the technical aspect, and the research gaps.

**Table 5:** Classification of 120 reviewed papers by thematic category.

Category	Description	Count	References
Statistical & Anomaly-Based	Early statistical threshold-based methods	13	Casas et al. (2012), García et al. (2014), Gulisano et al. (2015), Hoque et al. (2017), Jazi et al. (2017), Vidal et al. (2018), Jing et al. (2019), David and Thomas (2019), Prasad et al. (2020b), Çakmakçı et al. (2020), Bouyeddou et al. (2020), Rahman et al. (2024), Wang et al. (2025)
Network/ Application Layer	Application and network layer DoS detection	9	Stevanovic et al. (2013), Xiao et al. (2015), Nunes et al. (2017), Adi et al. (2017), Cambiaso et al. (2017), Hasan et al. (2018), Liang and Znati (2019), Park et al. (2020), Mohmand et al. (2022)
VoIP/Protocol-Specific	SIP and protocol-level attack detection	4	Sieklik et al. (2016), Dassouki et al. (2017), Semerci et al. (2018), Kurt et al. (2018)
Cloud-Based Detection	DoS/DDoS detection in cloud environments	30	Arshad et al. (2013), Vissers et al. (2014), Somani et al. (2016), Yadav (2019), Kanimozhi and Prem Jacob (2019), Patil et al. (2019), Aldribi et al. (2020), Rethishkumar and Vijayakumar (2020), Virupakshar et al. (2020), SaiSindhuTheja and Shyam (2021), Agarwal et al. (2021), Mohammad and Pradhan (2021), Karthick et al. (2022), Abdullayeva (2022), Sharma and Singh (2022), Agarwal et al. (2022), Chundru et al. (2022), Song (2023), Pasha et al. (2023), Hezavehi and Rahmani (2023), Farhat et al. (2023), Deniz and Serttaş (2023), Abdullayeva and Suleymanzade (2024), Pandithurai et al. (2024), Ouhssini et al. (2024), Shang (2024), Kumar and Agarwal (2024), Sharma and Malik (2025), Goyal et al. (2025), Selvy (2025)
SDN-Based Detection	Detection in software-defined networking environments	8	Wang et al. (2015), Shah et al. (2021), Reddy and Shyam (2022), Zhou et al. (2023), Ali et al. (2023a), Najar and Naik (2024), Bahashwan et al. (2025), Mehmood et al. (2025)
IoT/Edge/Fog Detection	Detection in distributed IoT and edge environments	14	Sharma et al. (2018), Kumar et al. (2021), Zhao et al. (2021), Sambangi et al. (2022), Reddy and Shyam (2022), Esmaeili et al. (2022), Priyadarshini and Barik (2022), Gaber et al. (2023), Yang et al. (2023), Lai et al. (2023), Sadhwani et al. (2023), Alani (2024), Binbusayyis (2024), Qureshi et al. (2025)
Ensemble & Hybrid ML/DL	Advanced hybrid and ensemble detection models	20	Kumar and Selvakumar (2013), Singh and De (2017), Hosseini and Azizi (2019), Garg et al. (2020), Prasad et al. (2020a), Kasım (2020), Thilagam and Aruna (2021), Kushwah and Ranga (2021), Geetha and Deepa (2022), Mayuranathan et al. (2022), Gowthul Alam and Michael Raj (2022), Yuan et al. (2023), Mustapha et al. (2023), Selvan et al. (2023), Islam et al. (2023), Balasubramaniam et al. (2023), Issa and Albayrak (2023), Shaikh et al. (2024), Abiramasundari and Ramaswamy (2025), Mandela and Etyang (2025)
Adaptive/Transfer Learning	Transfer and adaptive learning-based detection	7	Farzaneh et al. (2024), Bellmunt et al. (2025), Sun et al. (2026), Zhang et al. (2025), Ju et al. (2026), Ijaz et al. (2026), Chen and Ma (2026)
Blockchain & Emerging Security	Blockchain-integrated security frameworks	3	Kamal et al. (2022), Kotel et al. (2023), Jayakumari et al. (2024)
Survey/Systematic Review	Comprehensive review and survey papers	12	Mitchell and Chen (2014), Osanaiye et al. (2016), Singh et al. (2016), Iqbal et al. (2016), Coppolino et al. (2017), Khoda Parast et al. (2022), Lata and Singh (2022), Abdullayeva (2023), Ali et al. (2023b), Asha and Shanmugapriya (2024), Abdallah et al. (2024), Afraji et al. (2025)

In addition, the paper classification divides the detection papers based on whether they utilize the AI or ML technologies, whether the papers are non-AI detection papers using only statistical and rule-based approaches, or if they are survey/systematic review papers as detailed in Table 6. The proportionate distribution of the reviewed papers by their classification can also be seen in the pie chart depicted in Figure 2. It may be seen from Figure 2 that AI/ML-based detection constitutes the most popular approach in the existing literature, with the largest number of selected papers employing it. At the same time, the number of non-AI/ML detection methods used is much lower than that of AI/ML techniques.

**Table 6:** Corpus composition by paper type.

Paper classification	Count	Percentage of corpus
AI/ML-based Detection Papers	88	73.3%
Non-AI/ML Detection Papers	20	16.7%
Survey/Systematic Review Papers	12	10.0%
Total	120	100%

**Figure 2:**
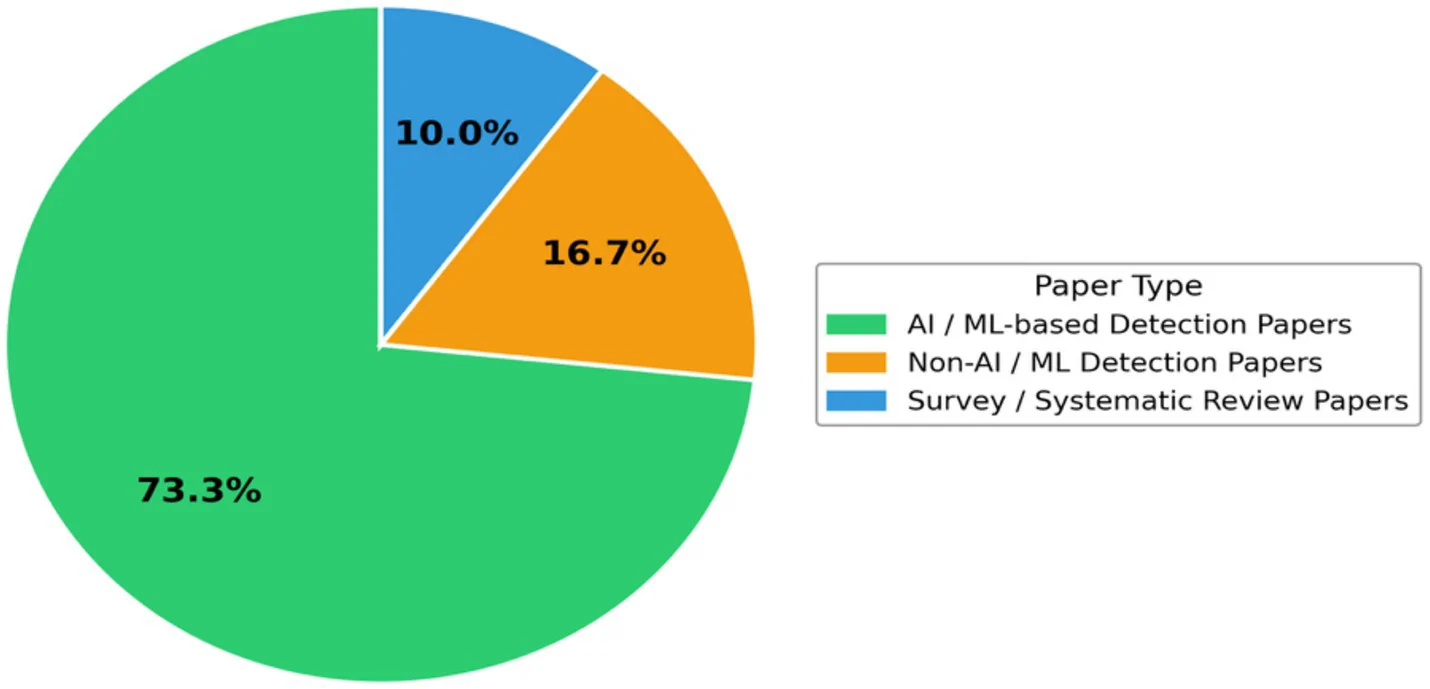
Pie-chart representation of paper type.

### 2.7 Temporal distribution of the corpus

The timeline chart of the 120 analyzed papers is indicative of the growing trend of research work on AI-enabled detection of DoS and DDoS attacks, as presented in Figure 3. The relatively low number of published articles between 2012 and 2015 is a consequence of an era when traditional statistical approaches to detecting network attacks were widely used. Starting from 2016, the growth of publications can be seen because of the use of machine learning approaches, combined with the availability of benchmark data sets that simulated actual attacks and the increased prevalence of cloud-based attack scenarios. A steep rise in the number of publications since 2022 up to 2026 demonstrates the growing interest in neural network models, hybrid approaches, and cloud-based detection models.

**Figure 3:**
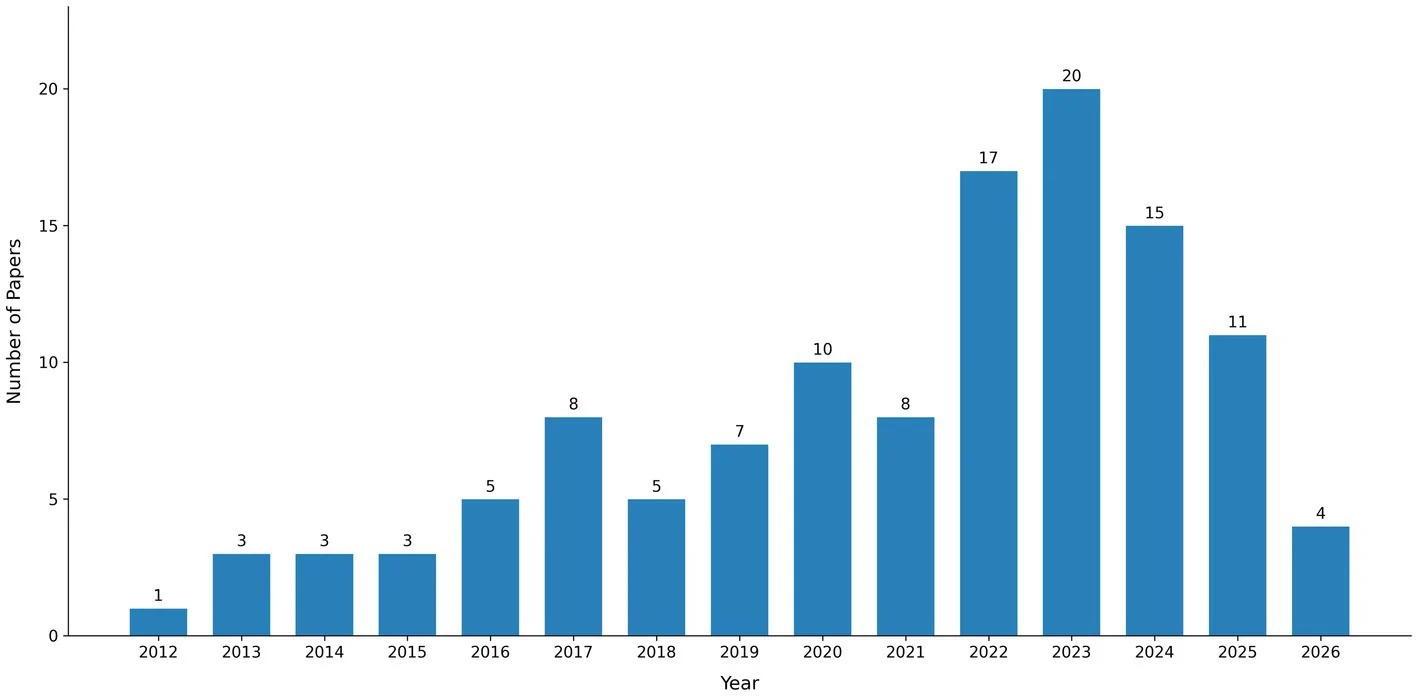
Year-Wise distribution of 120 reviewed papers from 2012 to 2026.

### 2.8 Data extraction

To ensure that the process of synthesis would be consistent and standardized for all 120 studies under examination, a standardized template for data extraction was adopted. The template covered the following variables: reference details including name of author, year of publication, and paper number; model/technique used in the study; study area; the dataset used; feature selection method; feature extraction technique; limitations of the paper; key findings; and results. The data extraction template facilitated the comparative analysis that was necessary for developing the comparative tables and performance assessment presented in later sections. The extracted data collectively forms the empirical foundation for the research gap analysis and future directions presented in Sections 8 and 9.

## 3 Literature review-evolution of DoS/DDoS detection

The high rate of change of Denial-of-Service (DoS) and Distributed Denial-of-Service (DDoS) attacks has led to the need to develop more advanced detection systems in different computing settings. The detection methods of early detection were based mainly on statistical analysis and threshold-based methods of anomaly detection. Nevertheless, as the attack patterns get more sophisticated, with the introduction of cloud computing, Software-Defined Networking (SDN), Internet of Things (IoT), and edge computing paradigms, researchers have suggested an extensive set of intelligent and adaptive detection strategies.

To conduct a systematic analysis of the development of these methods, this review classifies the literature on the subject according to the evolution of the DoS/DDoS detection mechanisms. The classification indicates the evolution of detection methods between the traditional statistical models to the present-day hybrid, transfer learning, and blockchain-based security systems. The studies reviewed can thus be classified into the following broad categories: statistical and early detection, network, and application-layer detection, protocol-specific detection, cloud-based detection, SDN-based detection, IoT, and edge-based detection, hybrid and ensemble models, transfer learning models, blockchain-based security, and survey studies.

### 3.1 Statistical and anomaly-based detection approaches

The earliest research in statistics-based and anomaly detection for DoS/DDoS focused mainly on unsupervised clustering and traffic analysis techniques, as illustrated by the works of Casas et al. (2012) and García et al. (2014). The latter researchers have proven that anomalies in the network traffic can be used to detect previously unknown attacks. To tackle the issue of scalability and real-time processing, later studies developed streaming and hardware-based detection techniques such as the work of Gulisano et al. (2015) and Hoque et al. (2017). Furthermore, adaptive entropy-based and dynamic thresholding approaches were utilized for more accurate detection of attacks in changing network conditions as shown by the contributions of Vidal et al. (2018) and David and Thomas (2019). In addition, further advancements were made using clustering algorithms, kernel learning, and measurement-driven statistical modeling as highlighted by the studies of Prasad et al. (2020b), Çakmakçı et al. (2020), Bouyeddou et al. (2020). Finally, the use of non-linear learning and cloud-based anomaly analysis was proposed to facilitate the adoption of intelligent and adaptive detection mechanisms by Rahman et al. (2024) and Wang et al. (2025). Detailed extraction data for statistical and anomaly-based detection studies are provided in Supplementary Table 1.

### 3.2 Network and application-layer detection

The shortcomings of statistical and anomaly-based detection techniques, especially the fact that they cannot detect mimicry-based and application-layer attacks, led to a research focus on network and application-layer detection based on intelligent learning models. This direction was first taken by Stevanovic et al. (2013), who used the unsupervised SOM and ART2 neural networks on logs of web servers and found that almost 52 percent of malicious crawlers imitated legitimate user behavior-a result which highlighted the fundamental insufficiency of purely statistical methods in application-layer threats. This spurred correlation-conscious and machine learning-inspired models, with Xiao et al. (2015) proposing CKNN-based flow correlation to detect data center DDoS attacks and Cambiaso et al. (2017) and Adi et al. (2017) concurrently developing the field by modeling stealthy slow DoS attacks and showing that SVM-based classifiers could identify them with zero false alarms. The field expanded further with Liang and Znati (2019) and Hasan et al. (2018), who comparatively tested intelligent methods with various types of traffic and extended deep convolutional detection to optical network scenarios, and Park et al. (2020) and Nunes et al. (2017) characterized the real-life constraints of the attacks and proposed autonomous multi-agent mitigation. The section ending was with Mohmand et al. (2022) whose ensemble-based classification on the UNSW-NB15 dataset heralded the definitive shift of the field to fully supervised and ensemble machine learning models to detect DoS/DDoS. Detailed extraction data for network and application-layer detection techniques studies are provided in Supplementary Table 2.

### 3.3 VoIP/protocol-specific attacks

Going beyond the generic network and application-layer detection frameworks, protocol-specific research also focused on the structural weaknesses of specific protocols that could not be sufficiently detected by generic detection frameworks. To define the magnitude of the legacy protocol exploitation, Sieklik et al. (2016) showed that approximately 60 × DDoS amplification is possible via TFTP servers, which opens a vulnerability of threats of a completely different nature compared to volumetric or behavioral anomalies. This inspired protocol-sensitive detection models, in which Dassouki et al. (2017) were able to demonstrate that both temporal and structural fingerprinting of SIP sessions could be used to reduce false positives to 0.24 percent-much lower than statistical thresholds - and Semerci et al. (2018) and Kurt et al. (2018) independently constructed unsupervised adaptive change point detection models in VoIP These works collectively indicated that protocol-specific structural awareness is, invariably, more effective than general statistical detection in detecting session-layer and amplification-based threats, and prepared the way to detection research in cloud infrastructures and their unique traffic space. Detailed extraction data for VoIP/protocol-specific attacks techniques studies are provided in Supplementary Table 3.

### 3.4 Cloud-based detection

While protocol-specific detection frameworks demonstrated that structural and session-level awareness significantly outperforms general statistical approaches for VoIP and amplification-based threats, the rapid cloudification of network infrastructure introduced an entirely new class of detection challenges rooted in virtualization, multi-tenancy, and distributed service architectures. Cloud-based DoS and DDoS detection research originated with early supervised and adaptive frameworks, wherein Arshad et al. (2013) established decision tree-based cloud intrusion classification, Vissers et al. (2014) introduced Gaussian-based adaptive outlier detection for application-layer DDoS in cloud web services, and Somani et al. (2016) empirically demonstrated that DDoS attack impose severe collateral infrastructure damage across non-targeted tenants in multi-tenant environments. It was followed by research extending to virtualization-layer defense mechanisms, with Yadav (2019) proposing hybrid fuzzy clustering using auto-associative neural networks, Rethishkumar and Vijayakumar (2020) introducing state transition modeling based on Markov Chains to co-resident DoS prevention, and both Aldribi et al. (2020) and Patil et al. (2019) Hyper-parameter optimized ANN was subsequently systematically developed as machine learning detectors by Kanimozhi and Prem Jacob (2019), ensemble tree models by Chundru et al. (2022) and Farhat et al. (2023), feature selection-based classification by Song (2023) and SaiSindhuTheja and Shyam (2021), and big data-integrated models by Mohammad and Pradhan (2021), while Virupakshar et al. (2020) and Deniz and Serttaş (2023) demonstrated deep learning superiority over classical approaches in OpenStack environments through DNN and LSTM architectures, respectively. Detection focused on low-rate and economically motivated attacks using several methods. Agarwal et al. (2021) used LSTM-based FRC detection. Pasha et al. (2023) developed a hybrid low-rate DDoS detection method. Hezavehi and Rahmani (2023) introduced IDARP-based anomaly detection. Pandithurai et al. (2024) applied a metaheuristic-optimized Bi-LSTM. Karthick et al. (2022) worked on behavioral neural detection. Abdullayeva (2022) employed unsupervised PCA-DBSCAN clustering. Agarwal et al. (2022) used WOA-DNN. Sharma and Singh (2022) focused on risk assessment modeling. Kumar and Agarwal (2024) implemented containerization-based defense. Finally, Sharma and Malik (2025) contributed with NLP-driven attack categorization. These efforts expanded cloud security beyond simple traffic classification. Shang (2024) showed that a Naive Bayes classifier trained on traffic captured by Wireshark can achieve about 99.78% detection accuracy in virtual cloud environments. This method outperformed Random Forest in prediction reliability. It confirmed that lightweight probabilistic models can be effective even alongside more complex architectures. The section concludes with significant contributions. These include graph neural network-based detection by Abdullayeva and Suleymanzade (2024), dual-attention BiGRU by Goyal et al. (2025), transformer-augmented DeepDefend by Ouhssini et al. (2024), and optimization-driven temporal inductive path neural networks by Selvy (2025). This represents a major shift in cloud DoS and DDoS detection. We move from static decision tree methods to attention-based, graph-structured, and transformer-enhanced deep learning approaches. This progress lays the groundwork for the SDN-based programmable detection frameworks discussed in the next section. Detailed extraction data for cloud-based detection techniques studies are provided in Supplementary Table 4.

### 3.5 SDN-based detection

The simultaneous advent of Software-Defined Networking (SDN) presented a radically new paradigm of detection based on programmable visibility of control-plane, centralized flow control, and adaptive policy control. Wang et al. (2015) pioneered this research direction by the DaMask graphical probabilistic inference model with 89.30% detection accuracy on the UNB ISCX data in a hybrid SDN-cloud setting. Shah et al. (2021) and Reddy and Shyam (2022) then discussed the use of economically motivated flooding threats through the EDOS-IDM and EDOS-TSM models using the statistical anomaly detection, binomial probability estimation, and TTL analysis to counter ICMP and TCP SYN-based EDoS attacks in OpenStack SDN deployments with close to zero residual attack bandwidth. Detection capability was subsequently improved by using distributed and deep learning models, with Zhou et al. (2023) reporting 96.07% accuracy on five benchmark datasets by using CoWatch collaborative edge-SDN framework, and Ali et al. (2023a) showing systematic comparative analysis that SVM yields the most favorable accuracy-efficiency trade-off (95.57%, F1: 0.97) among the most recent works, such as CNN-based detection by Najar and Naik (2024) with 99.99% binary classification accuracy, MLP-based SDN controller defence by Bahashwan et al. (2025) with 98.068% accuracy, and optimizer-based hybrid CNN-MLP by Mehmood et al. (2025) with 99.98% accuracy. Detailed extraction data for network and SDN-based detection techniques studies are provided in Supplementary Table 5.

### 3.6 IoT, fog, and edge-based detection

Although the studies on SDN-based intrusion detection systems have confirmed that highly accurate classification could be achieved with the help of a programmable control plane architecture along with the use of hybrid deep learning algorithms, the increased usage of IoT devices, which operate in a decentralized manner, brought forward new challenges regarding detection based on resource constraints, heterogeneous traffic, and decentralized architecture. The research area related to these problems emerged from DBN-based studies where, according to the contributions of Sharma et al. (2018) and Reddy and Shyam (2022), the cloud-SDN and SaaS DBN frameworks could be regarded as the foundations of the detection algorithms in IoT. As time went on, the detection techniques improved with more complex structures based on ensembles and feature engineering, as seen with the works of Kumar et al. (2021), Sambangi et al. (2022), Sadhwani et al. (2023), which indicated that stacking-based ensemble models, Gaussian similarity feature transformation, and feature-selected classifier approaches enhanced detection capability in different IoT traffic. Lightweight and adaptive algorithms suitable for constrained environments emerged with the contributions of Zhao et al. (2021) and Lai et al. (2023), which provided decision tree-based edge detection for IoT traffic and online passive-aggressive machine learning approach for WSNs, respectively. The utilization of deep learning models was continued with the parallel recurrent structure where Priyadarshini and Barik (2022), Esmaeili et al. (2022), Farzaneh et al. (2024) gradually advanced LSTM and BiLSTM-based detection algorithms from fog-SDN environment to transfer learning and 5G network slicing. Hybrid convolutional and recurrent architectures were investigated by Binbusayyis (2024) and Qureshi et al. (2025), utilizing VGG19-2D-CNN and LSTM-GRU in fog-cloud and industrial IoT environment, respectively, and Gaber et al. (2023), Yang et al. (2023), Alani (2024) made domain-specific contributions toward detection in vehicular, industrial CPS, and smart city IoT infrastructures with SDN-based honeypots. Detailed extraction data for network and IoT, fog and Edge-based detection techniques studies are provided in Supplementary Table 6.

### 3.7 Ensemble and hybrid ML/DL-based detection

Although IoT, edge- and fog-based detection models demonstrated that distributed and hybrid deep learning systems are suitable to tackle resource-constrained network threats, research on ensemble and hybrid ML/DL models developed concurrently to develop detection intelligence via algorithmic fusion, metaheuristic optimization, and architectural hybridization. This line of research was pioneered by Kumar and Selvakumar (2013) with NFBoost-neuro-fuzzy boosting ensemble, which has reached a very high level of accuracy (about 99 percent) on a variety of datasets, and by Singh and De (2017), who simultaneously showed that genetic algorithm-optimized MLP (MLP-GA) is much more effective at application-layer DDoS detection than traditional. Later work expanded on structured approaches to crafting features and combining models. Notably, Hosseini and Azizi (2019) built a combined Random Forest setup using client-proxy design, reaching an F-measure of 99.7% on NSL-KDD data. In another direction, Garg et al. (2020) formed the En-ABC system, merging Restricted Boltzmann Machines with clustering based on bee behavior, which scored 97.62% correct classifications. Meanwhile, Prasad et al. (2020a) improved threat identification by applying Bayesian-Rough logic along with probability-based attribute filtering within the CICIDS2017 dataset. Elsewhere, Kushwah and Ranga (2021) showed how evolving neural networks-called (SaE-ELM-Ca) could hit near-perfect accuracy at 99.99% on identical test conditions. Together, these results underline how careful choice of inputs paired with tuned algorithms cuts down incorrect alerts when tested across standard evaluation sets. After that, smarter ways of tuning models took hold. Kasım (2020), using autoencoders tied to SVM, hit 99.41% correct predictions on CICIDS2017. Instead of traditional methods, Thilagam and Aruna (2021) turned to Ant Lion tricks to guide an RC-NN for spotting cloud threats. A different path came from Mayuranathan et al. (2022), who built a Deep Kronecker setup sharpened by chaotic deer movements-landing at 97.22% accuracy. Then, Gowthul Alam and Michael Raj (2022) pushed SVM further, boosted by Elephant Herds moving in discrete steps, reaching nearly perfect marks: 99.99%. Meanwhile, work by Balasubramaniam et al. (2023) showed stacked encoders tuned by GHLBO managing 91.7% accuracy across two datasets, NSL-KDD plus BOT-IoT. Among recent efforts, Mustapha et al. (2023) applied an adversarial WGAN-LSTM method that reached a 0.99 F1-score using the CIC-DDoS2019 dataset; meanwhile, detection tactics shifted toward optimized neuro-fuzzy systems when Selvan et al. (2023) tested their Fractional Anti-Corona Virus Optimization approach on NSL-KDD and BOT-IoT. With fusion methods gaining attention, Yuan et al. (2023) built the AMLFN-AD structure - delivering 99.32% accuracy within NSL-KDD experiments. Rather than relying solely on deep models, Islam et al. (2023) combined SVM, KNN, and Logistic Regression to counter Memory DoS threats, reporting 96% accuracy. Detection limits expanded further under Geetha and Deepa (2022), who enhanced BiLSTM performance via Fisher Kernel PCA paired with Grey Wolf Optimization, yielding near-perfect results over four standard benchmarks datasets. The section culminates with hybrid CNN-LSTM by Issa and Albayrak (2023) achieving 99.31% accuracy on NSL-KDD, PCA-driven Random Forest by Abiramasundari and Ramaswamy (2025) attaining 98.9% accuracy across CIC datasets, RNN-based comparative evaluation by Mandela and Etyang (2025) establishing sequence models as superior for temporal traffic pattern capture on CIC-DDoS2019, and CNN-Vision Transformer hybridization by Shaikh et al. (2024) collectively marking the progression of ensemble and hybrid detection from neuro-fuzzy boosting baselines toward transformer-augmented, optimization-driven deep learning architectures for advanced network intrusion detection. Detailed extraction data for Ensemble & Hybrid ML /DL-based detection techniques studies are provided in Supplementary Table 7.

### 3.8 Meta−/ adaptive−/ transfer-learning approach-based detection

Further beyond the limited scope of static detector architectures, Farzaneh et al. (2024) proposed DTL-5G, a deep transfer learning system to detect DDoS in 5G network slicing settings, which is based on BiLSTM and trained on a six-million-record testbed dataset and tested against the publicly available 5G-NIDD benchmark. DTL-1 transfer strategy with 94.87 and 96.69 percent accuracy and recall even a 13.90 and 21.48 percent improvement over the baseline. It shows that knowledge transfer across large source domains effectively overcomes the issue of label scarcity in emerging 5G infrastructures and makes deep transfer learning a feasible adaptive detection paradigm in next-generation programmable networks. Detailed extraction data for Meta−/ Adaptive−/ Transfer-Learning Approach-based detection techniques studies are provided in Supplementary Table 8.

### 3.9 Blockchain and emerging security

The protection paradigm expanded to cover data integrity, decentralized trust, and cryptographic security of cloud infrastructures by blockchain and new research on security, no longer depending on traffic-based intrusion detection. The direction has been laid down by Kamal et al. (2022), who set the base by setting up a Privacy-aware Genetic Algorithm-based tool which combines intelligent encryption along with parallel distributed cloud storage achieving 17.06 percent reduction in execution time and 21.11 percent throughput over AES, which proves that, genetic algorithm-driven selective encryption is a computationally effective alternative to traditional cryptographic solutions to provide large-scale cloud data confidentiality. Later studies shifted from cryptographic encryption toward decentralized architectural security frameworks. To minimize transparency, non-repudiation, and attack resistance in IoT smart home settings, Kotel et al. (2023) recommend using a permissioned blockchain platform, Hyperledger Fabric based in four integrated layers that are not dependent on ML-based classification. Further developing this decentralized base, Jayakumari et al. (2024) brought blockchain security to cloud-civic infrastructure as a hybrid blockchain E-voting system with ECDSA digital signatures, smart contracts and PBFT consensus protocols-achieving more than 50 percent savings in authentication delays, and around 55 percent reduction in vote-modification rate-laying the groundwork of the emergent cloud security from cryptographic baselines toward consensus-driven blockchain architectures capable of securing both IoT ecosystems and mission-critical cloud applications. Detailed extraction data for Blockchain & Emerging Security techniques studies are provided in Supplementary Table 9.

### 3.10 Survey/systematic review

Though blockchain along with new security models brought decentralized trust and encryption into alignment with machine learning detection methods, reviews in academic journals performed an equivalent but distinct function - pulling together dispersed findings, exposing missing categories, and shaping what comes next in cloud and network safety studies. Early groundwork appeared in work by Mitchell and Chen (2014), focused on wireless intrusion systems, alongside Osanaiye et al. (2016), who examined resistance to cloud-based DDoS attacks; later contributions emerged through layered system classifications from Singh et al. (2016), service-oriented designs detailed by Iqbal et al. (2016), plus coordinated defences across networks, hypervisors, and hardware outlined in Coppolino et al. (2017). With advancements in the study area, surveys became more focused on consolidating knowledge within specific fields. DoS/DDoS attacks and shared virtualization were found to be the most important recurring threats in both IaaS, PaaS, and SaaS architectures by Khoda Parast et al. (2022). Additionally, Lata and Singh (2022) recommended hybrid IDS systems with feature selection for scalable intrusion detection for clouds, and Abdullayeva (2023) suggested a reference framework for cyber resilience based on confidentiality, integrity, availability, and fault tolerance across all cloud and IoT layers. The latest research efforts focus on integrating ML and DL techniques within specific threat domains. According to Ali et al. (2023b), DL algorithms prove to be more effective than traditional ML algorithms in detecting DDoS attacks in SDN environments, with a near-perfect accuracy of about 99.94%. Meanwhile, Abdallah et al. (2024) and Afraji et al. (2025) highlight scalability, data heterogeneity, and explainability as three key unresolved issues in cloud anomaly detection and DDoS mitigation. Furthermore, Asha and Shanmugapriya (2024) stress the lack of suitable datasets to support the detection of insider threats in clouds. From this analysis, one may conclude that while ML and DL technologies are quite well-developed, further cloud security research requires the development of standardized evaluation methodologies and the use of explainable AI approaches. Detailed extraction data for Survey/ Systematic Review studies are provided in Supplementary Table 10.

## 4 Feature engineering techniques

The role of feature engineering in DoS and DDoS detection is vital in setting the quality and discriminative capabilities of input representations to detection models. Throughout the 120 studies reviewed, feature engineering methods have developed over the years since the inception of manual traffic feature extraction in early statistical models, to fully automated deep feature learning in modern architectures, and multiple paradigms have been found to dominate the corpus.

### 4.1 Statistical and flow-based feature extraction

The most basic method is based on statistical and flow-based feature extraction of raw network packets or NetFlow records. Early papers by Casas et al. (2012), Gulisano et al. (2015), and García et al. (2014) used direct packet counts, the volume of bytes, the duration of flows and IP entropy directly out of the traffic traces without explicit selection. CICFlowMeter then became the most used automated extraction tool in post-2018 research-used by Kanimozhi and Prem Jacob (2019), Mayuranathan et al. (2022), Abiramasundari and Ramaswamy (2025), Ouhssini et al. (2024) creating 80–84 statistical traffic features that are then filtered with selection mechanisms. This convergence of flow feature extraction by CICFlowMeter is a notable convergence of a methodology in cloud, SDN and hybrid detection literature since 2019.

### 4.2 Filter-based feature selection

The most frequently used selection paradigm in the reviewed corpus was filter-based selection based on statistical measures that are not dependent on detection models. Liang and Znati (2019) used Information Gain and Gain Ratio to rank packet-header features and Najar and Naik (2024) used it to CNN-based SDN detection. Song (2023) used chi-square testing and information gain on three benchmark datasets with an accuracy of 99.993%. Mustapha et al. (2023) used Pearson correlation, mutual information, and mRMR to do adversarial WGAN-LSTM detection, and Lai et al. (2023) used filter-based selection as their GA-MIC-based extension of filter-based statistical measures, as the baseline feature selection methodology in network traffic classification tasks.

### 4.3 Wrapper-based and metaheuristic feature selection

Wrapper-based feature selection and metaheuristic-driven feature selection offered significantly better detection performance at a greater cost. A binary bat algorithm by Patil et al. (2019) was the Extended Binary Bat Algorithm that selected 26 optimized flow-based features with 99.51% accuracy and 0.04% FPR in a real cloud testbed. KPCA has been used in conjunction with Discrete Elephant Herding Optimization (DEHO) by Gowthul Alam and Michael Raj (2022) with an accuracy of 99.99 on CIC-IDS2017. SaiSindhuTheja and Shyam (2021) introduced the Oppositional Crow Search Algorithm (OCSA), which on KDD Cup 99 had 98.18% precision, Honey Badger Optimization with 97% accuracy (Pandithurai et al., 2024). Bi-LSTM accuracy and Brown Bear Optimization Algorithm (BBOA) with 99.8% accuracy on CSE-CIC-IDS2018 (Selvy, 2025). Together, these independent experiments validate that nature-inspired optimization is significantly superior in terms of high-dimensional cloud and IoT detection tasks, compared to manual and filter-based feature selection, but with a substantial increase in training complexity.

### 4.4 Dimensionality reduction

The most popular dimensionality reduction method was Principal Component Analysis (PCA), which was used independently by Abdullayeva (2022) to unsupervised DBSCAN-based cloud DDoS detection, Abiramasundari and Ramaswamy (2025) to detect with Random Forest across various CIC datasets, and Shaikh et al. (2024) in combination with SMOTE to detect with hybrid CNN-Vision Transformer Fisher Kernel PCA (FKPCA) with Grey Wolf Optimization by Geetha and Deepa (2022) showed that nonlinear reduction kernel-based reduction is more effective at preserving attack-discriminative variance in high-dimensional spaces of traffic features than linear PCA. SVD with Jensen-Shannon divergence and Fractional Distribution Change by Wang et al. (2025) was the most advanced dimensionality reduction application to the corpus. The method used in network-layer traffic patterns applied to service-layer performance distributions of cloud API runtime reliability anomalies.

### 4.5 Deep automated feature learning

A large percentage of deep learning-based studies did not explicitly select features, instead, learning neural features automatically. Hasan et al. (2018) used CNN-based spatial feature extraction to detect OBS network BHP floods, Virupakshar et al. (2020) to detect OpenStack DDoS floods, and Najar and Naik (2024) to detect SDN floods where convolutional layers learned representations based on traffic patterns directly on raw feature matrices. Autoencoder-based latent representation by Kasım (2020) down sampled the 80-feature CICFlowMeter space to a smaller representation which was further classified by SVM with 99.41% accuracy on CICIDS2017. Priyadarshini and Barik (2022) and Agarwal et al. (2021) using Discrete Wavelet Transform preprocessing, and Farzaneh et al. (2024) used LSTM and BiLSTM temporal feature learning in 5G environments with transfer learning. Graph topology feature vectors of the graph topology feature vectors (Abdullayeva and Suleymanzade, 2024) used graph topology feature vectors defined a multi-flow relational dependence that is not included in the conventional feature vectors. The best architecturally developed one was the Dual Attention Dense BiGRU with Improved Cheetah Optimization by Goyal et al. (2025), which does both selection and deep feature fusion in five benchmark datasets with better accuracy.

### 4.6 Entropy-based and probabilistic feature engineering

Entropy-based feature engineering was a domain-specific paradigm that was eminent in statistical and protocol-specific detection research. Vidal et al. (2018) used the variation of Rényi entropy using HoltWinters time-forecasting as the main detection signal in KDD’99, CAIDA’07, CAIDA’08 and real campus traffic. Jing et al. (2019) integrated entropy-based flow features with MM-CUSUM change-point statistics with MAWI and CIC-IDS2017. Ouhssini et al. (2024) used CNN-LSTM-Transformer entropy forecasting as a preprocess signal to only activate deep detection when the entropy showed an anomaly limiting active Compute to less than 1% of monitoring time yet retaining 99.97% accuracy. Wang et al. (2025) used SVD using fractional distribution change and JensenShannon divergence as probabilistic feature signals to identify anomalies in cloud service API reliability - the entropy-based feature engineering of network-layer traffic distributions to service-layer performance distributions.

### 4.7 Domain-specific and protocol-aware feature engineering

Some of the studies constructed feature engineering depending on the type of attack or environment. Dassouki et al. (2017) generated temporal and structural SIP message fingerprints to detect cloud-based VoIP flooding, with 0.24% FPR and 1.95 s to detect a session-layer attack-on-network, which shows protocol-specific structural characteristics are a significant improvement over generic flow statistics to differentiate session-layer attacks. Jazi et al. (2017) elicited HTTP session-level features to detect CUSUM-based application-layer DoS, and found that sampling strategy badly distorts feature distributions, and decreases detection rates by 100 to 88.46% with sketch-guided 30% sampling. Zhao et al. (2021) created multimedia specific protocol and session analysis functions to identify edge IoT traffic with 98% image traffic accuracy. Sharma and Malik (2025) used Word2Vec embeddings and LSTM sequential feature learning to NLP-based cloud threat text corpora to categories attacks in IaaS, PaaS, and SaaS service models the only study in the reviewed corpus that used natural language processing as the main feature engineering paradigm to classify cloud security threats.

### 4.8 Summary and key observations

The evolution of feature engineering in the literature reviewed is summarized by three cross-cutting trends. First, the obvious shift between manual extraction (Casas et al., 2012; García et al., 2014; Gulisano et al., 2015) to filter-based (Liang and Znati, 2019; Song, 2023; Lai et al., 2023) and metaheuristic selection (Patil et al., 2019; SaiSindhuTheja and Shyam, 2021; Gowthul Alam and Michael Raj, 2022; Pandithurai et al., 2024; Selvy, 2025) to automated deep feature learning (Hasan et al., 2018; Kasım, 2020; Abdullayeva and Suleymanzade, 2024; Goyal et al., 2025) is an indication of the overall move of the field toward data-driven rather than knowledge-driven detection paradigms. Second, CICFlowMeter is now the default flow feature extraction tool in post-2018 papers, establishing a methodological consistency, but also a dependency on widely available open-source tools that might not be generalizable to other network contexts and attack types. Third, and most importantly, the lack of feature interpretability among metaheuristic and deep learning-based models, whereby selected features do not bring about any explainable justification of detection decisions, is a direct driver to integrate SHAP-based and attention-based explainable feature importance mechanisms as a top priority future research direction, as confirmed by survey responses of Abdallah et al. (2024) and Afraji et al. (2025).

## 5 Evaluation metrics

To measure and compare DoS and DDoS detection systems, it is critical to have consistent and complete evaluation metrics. In the 120 studies reviewed, a wide and frequently conflicting range of metrics was used-starting with simple accuracy in earlier statistical models, to multi-dimensional measures of performance in modern deep learning models. This part examines the most prevalent measures of evaluation embraced throughout the corpus and pinpoints a methodological gap in the form of metric inconsistency.

### 5.1 Accuracy

The most reported measures in all the ten detection domains are accuracy, with high scores of 99.97% by Kanimozhi and Prem Jacob (2019), 99.9% at (Chundru et al., 2022), 99.99% at (Najar and Naik, 2024), and 99.8% at (Selvy, 2025) being the highest. But accuracy as a single measure of intrusion detection is fundamentally unsatisfactory in the conditions of extreme class imbalance, i.e., where a classifier predicts all examples as normal, with accuracy then giving the misleading impression of high performance whilst classifying no attacks, which is the reason why precision and recall, along with F1-score, are complementary measures of quality.

### 5.2 Precision, recall, and F1-score

The most informative evaluation triad of intrusion detection is precision, recall, and F1-score which are used in most of the reviewed ML and DL papers. Precision measures burden of false alarms - Hosseini and Azizi (2019) obtain 99.7% F-measure, and Patil et al. (2019) obtain 0.04% FPR. Recall measures detection completeness-Kumar et al. (2021) (99.98%) and Bouyeddou et al. (2020) on DARPA99 have 100% TPR. The harmonic mean of precision and recall, F1-score, has increasingly replaced standalone accuracy as the primary metric in recent work, with reported values of Gaber et al. (2023) at 99.58%, Mustapha et al. (2023) at 0.99 F1-score and Goyal et al. (2025) at 98.5% across five datasets, indicative of an increasing methodological maturity in cloud, IoT and hybrid detection research.

### 5.3 False positive rate and false negative rate

FPR and FNR are operationally relevant measures of alert fatigue and unanticipated attack risk, respectively. FPR is often reported in statistical studies (Casas et al., 2012) below 3.5%, Bouyeddou et al. (2020) as low as 0.18 and 0.23% on CICIDS2017. FNR is significantly less covered with the leading result of 0.0059% in LSTM-based FRC detection in (Agarwal et al., 2021) and 0.004% in FPGA-based detection in (Hoque et al., 2017) respectively. The systematic under reporting of FNR through the corpus is an important gap in the evaluation since the risk of missed attacks is significantly more operationally security-critical than false alarms in most network settings.

### 5.4 Area under the ROC curve

AUC-ROC is a threshold-free measure of discrimination that has been reported in a subset of studies reviewed, Kanimozhi and Prem Jacob (2019) at 0.999, Farhat et al. (2023) at 99.94% using XGBoost, Kasım (2020) at 0.9988 on virtual DDoS traffic, and Najar and Naik (2024) at 1.00 for binary CNN-based SDN detection. AUC is best used when there are several classifiers at different thresholds as shown by Rahman et al. (2024) at 0.997 on UNSW-NB15. Its relative lack of use in the literature of both the IoT and protocol-specific work may reflect domain specificity rather than methodological neglect, though this interpretation requires further investigation.

### 5.5 Matthews correlation coefficient

MCC is a balanced binary classification quality measure that is resistant to class imbalance, reported in a small but increasing number of studies - Ali et al. (2023a) at 0.94 on SVM-based SDN detection, Geetha and Deepa (2022) at up to 0.84 on four benchmark datasets, and Selvy (2025) at 0.99 on CICDDoS2019. MCC is unpopular in the reviewed corpus despite its theoretical superiority over F1-score on imbalanced classification, and this methodological gap in detection studies in the future should focus especially on the highly imbalanced datasets such as CICIDS2017 and BoT-IoT.

### 5.6 Detection latency and computational efficiency

*Latency of detection*: Operational deployment is sensitive to detection latency but not consistently reported. Hoque et al. (2017) reported the lowest detection latency in the corpus at less than 1 microsecond with FPGA implementation - the lowest recorded. Alani (2024) reported inference time per packet of 0.035 μs, Patil et al. (2019) 3.7 microseconds per connection, Qureshi et al. (2025) about 6 ms hybrid LSTM-GRU IIoT detection and Ouhssini et al. (2024) reduced active computational overhead to less than 1% of monitoring time and retained only a fraction of active processing overhead. The systematic latency reporting gap in most cloud and ensemble experiments determined solely by offline benchmark accuracy-is a gap between experimental and operational deploy ability.

### 5.7 Metric inconsistency and implications

Analysis in cross study indicates that there is a great deal of inconsistency in metrics which essentially constrain comparability throughout the corpus. Early statistical studies report TPR and FPR (Casas et al., 2012; García et al., 2014; Gulisano et al., 2015; Hoque et al., 2017; Jazi et al., 2017; Vidal et al., 2018; Jing et al., 2019; David and Thomas, 2019; Bouyeddou et al., 2020), ML-based studies favor accuracy, precision, recall, and F1-score (Xiao et al., 2015; Adi et al., 2017; Singh and De, 2017; Liang and Znati, 2019; Hosseini and Azizi, 2019; Patil et al., 2019; Mohmand et al., 2022; Chundru et al., 2022; Farhat et al., 2023; Sadhwani et al., 2023; Abiramasundari and Ramaswamy, 2025), deep learning studies increasingly adopt AUC and MCC (Kanimozhi and Prem Jacob, 2019; Kasım, 2020; Ali et al., 2023a; Najar and Naik, 2024; Rahman et al., 2024; Selvy, 2025), while protocol-specific studies report domain-specific metrics such as detection time and amplification factor (Sieklik et al., 2016; Hoque et al., 2017; Ouhssini et al., 2024; Alani, 2024). García et al. (2014), Ali et al. (2023b), Abdallah et al. (2024), Afraji et al. (2025) are among the first to contribute to the list of survey results that pinpoint the inconsistency as one of the greatest impediments to reproducible evaluation in the field. The lack of a standardized assessment procedure that defines a minimum acceptable number of metrics (such as accuracy, F1-score, FPR, FNR, AUC, and detection latency on standardized benchmarks) is a critical methodological gap that needs to be closed by future systematic studies to allow meaningful cross-study comparisons and evidence-based adoption of detection frameworks to operational deployment.

## 6 Datasets used in literature

The effectiveness and applicability of any intrusion detection or DDoS mitigation structure are highly dependent on the testing data sets. The comprehensive examination of the literature reviewed in this article reveals the evident change in the use of datasets. It has gone beyond the early simulated laboratory settings, like KDD99 and DARPA, to large scale and diverse and application specific datasets, such as CIC-IDS2017, UNSW-NB15 and CIC-DDoS2019. Such more recent data sets are more reflective of the current attack trends. Table 15 lists all the datasets found in the studies reviewed, their references, links to access, and names.

### 6.1 Legacy datasets

This was the first standardized intrusion detection test set, KDD Cup 1999 (KDD99) (Casas et al., 2012; Kumar and Selvakumar, 2013; Xiao et al., 2015; Osanaiye et al., 2016; Vidal et al., 2018; Prasad et al., 2020b; SaiSindhuTheja and Shyam, 2021; Lata and Singh, 2022; Ali et al., 2023b), published in 9 studies but with well-publicized limitations, including redundant records, grossly unequal classes and lack of current attacks. Its refined successor, NSL-KDD (Hosseini and Azizi, 2019; Garg et al., 2020; Kasım, 2020; Kushwah and Ranga, 2021; Zhao et al., 2021; Geetha and Deepa, 2022; Karthick et al., 2022; Gowthul Alam and Michael Raj, 2022; Abdullayeva, 2022; Lata and Singh, 2022; Esmaeili et al., 2022; Yuan et al., 2023; Selvan et al., 2023; Ali et al., 2023b; Balasubramaniam et al., 2023; Issa and Albayrak, 2023; Goyal et al., 2025), emerged as the second most frequently adopted dataset (17 studies) by resolving these structural deficiencies, though both remain unable to represent contemporary network environments. The DARPA 1998/1999 evaluation data (Osanaiye et al., 2016; Hoque et al., 2017; Liang and Znati, 2019; David and Thomas, 2019; Bouyeddou et al., 2020; Thilagam and Aruna, 2021; Mayuranathan et al., 2022) data (7 studies) and the MAWI backbone traffic data (Casas et al., 2012; Xiao et al., 2015; Jing et al., 2019; Bouyeddou et al., 2020) data (4 studies) have a similar reflective nature of foundational, but more dated contributions, where MAWI has unlabeled real traffic suited specifically for anomaly detection research rather than supervised classification.

### 6.2 CIC and UNSW dataset families

The majority of the experimental evaluations in the reviewed corpus are based on datasets from the Canadian Institute for Cybersecurity and the UNSW Canberra Cyber Range. NSL-KDD is the most frequently used dataset in 17 studies, followed by KDD Cup 99 which is used in 9 studies, due to its balanced class distribution and attack coverage, superior to that of its predecessor KDD Cup 99. CICIDS2017 is followed by 19 studies, which is designed with realistic traffic generation methodology with multi-attack taxonomy covering DoS, DDoS, Brute-force, Botnet and Web attack categories. The benchmark is expanded with the inclusion of a wider set of modern attack scenarios from a larger network infrastructure, CSE-CIC-IDS2018. CSE-CIC-IDS2018 is used in 15 studies, extending the CICIDS2017 benchmark with a broader range of modern attack scenarios captured across a larger network infrastructure. In the current DDoS attack vectors such as SYN, NTP, DNS and LDAP floods, CICDDoS2019 is mentioned in 10 studies specifically involving DDoS classification. UNSW family family, UNSW-NB15 is used in 12 studies, and has been valued for its nine distinct attack categories, and for its real-world network traffic profiles, which better capture realistic network behavior than does the traffic from the DARPA era. The former, BoT-IoT is encountered in 8 studies, and the latter, TON-IoT, is growing in popularity for benchmarking IoT-related security assessments, especially for edge and IoT detection frameworks. The ISCX IDS 2012 is mentioned in 9 studies and is a transitional dataset between the legacy DARPA datasets (mentioned in 7 studies) and the modern CIC family. There are 7 studies that mention CAIDA DDoS 2007, most of which are statistical and correlation-based detection studies. A few of the studies in the corpus use MAWI and InSDN, which represent the small, but increasing, number of SDN-specific benchmarks.

### 6.3 Specialized and SDN-specified datasets

The CAIDA DDoS Attack 2007 dataset (Kumar and Selvakumar, 2013; Gulisano et al., 2015; Osanaiye et al., 2016; Hoque et al., 2017; Singh and De, 2017; Vidal et al., 2018; Liang and Znati, 2019) (7 studies) offers one hour of anonymized pcap traces of a real-world DDoS attack and has high-fidelity attack traffic but limited normal-traffic coverage - limiting standalone training of classifiers. InSDN dataset (Alani, 2024; Mehmood et al., 2025) (2 studies) (introduced by Elsayed et al. (2020), using IEEE Access) is used to fill the gap of SDN-compatible labeled benchmarks with 343,939 instances of DoS, DDoS, web attacks, and exploitation in an OpenFlow-based testbed. Domain-specific datasets such as TUIDS (Hoque et al., 2017; Geetha and Deepa, 2022) (Tezpur University; 2 studies) and custom experimental generated corpora, such as Slowloris traffic, VANET NS2 simulation traces, and industrial ICS testbed captures, all reflect the current lack of publicly available, labeled benchmarks of new attack vectors and deployment settings.

### 6.4 Adoption patterns and implications

There are three patterns that are evident through a cross-sectional analysis. First, the adoption of benchmarks is still highly focused, with the two benchmark options described above - CIC-IDS2017 and NSL-KDD alone are cited 35 times cumulatively - suggesting that comparative analysis might not be as generalizable as possible to heterogeneous settings. Second, dataset IoT-specific (ToN-IoT, BoT-IoT) are underrepresented when compared to the growth of attack research focused on IoT, which highlights the necessity to further develop domain-specific benchmarks. Third, the corpus simply does not include SDN- and 5G-related datasets, with InSDN being the only SDN benchmark, which is also an issue that should be addressed as soon as possible as programmable and next-generation networks are rolled out. The next step in research should focus on benchmark diversification, i.e., encrypted traffic, zero-day attack patterns, and multi-domain IoT environment, to enhance the validity of detection framework evaluation in a real-world application. Summary of benchmark datasets used in this review is mentioned in Table 7 and represented as bar chart in Figure 4.

**Table 7:** Consolidated summary of benchmark datasets in reviewed literature.

Dataset	References	Official Link	Notes
KDD99/KDD Cup 1999 (Dua and Du, 2016)	Casas et al. (2012), Kumar and Selvakumar (2013), Xiao et al. (2015), Osanaiye et al. (2016), Vidal et al. (2018), Prasad et al. (2020b), SaiSindhuTheja and Shyam (2021), Lata and Singh (2022), Ali et al. (2023b)	https://kdd.ics.uci.edu/databases/kddcup99/kddcup99.html	Superseded by NSL-KDD; redundancy & imbalance issues.
NSL-KDD Tavallaee et al. (2009)	Hosseini and Azizi (2019), Garg et al. (2020), Kasım (2020), Kushwah and Ranga (2021), Zhao et al. (2021), Geetha and Deepa (2022), Karthick et al. (2022), Gowthul Alam and Michael Raj (2022), Abdullayeva (2022), Lata and Singh (2022), Esmaeili et al. (2022), Yuan et al. (2023), Selvan et al. (2023), Ali et al. (2023b), Balasubramaniam et al. (2023), Issa and Albayrak (2023), Goyal et al. (2025)	https://www.unb.ca/cic/datasets/nsl.html	Refined KDD99 variant. Hosted by CIC, Univ. of New Brunswick.
DARPA 1998/1999 DARPA (2000)	Osanaiye et al. (2016), Hoque et al. (2017), Liang and Znati (2019), David and Thomas (2019), Bouyeddou et al. (2020), Thilagam and Aruna (2021), Mayuranathan et al. (2022)	https://www.ll.mit.edu/r-d/datasets/1999-darpa-intrusion-detection-evaluation-dataset	MIT Lincoln Laboratory. Foundational but dated.
MAWI Fontugne et al. (2010)	Casas et al. (2012), Xiao et al. (2015), Jing et al. (2019), Bouyeddou et al. (2020)	https://mawi.wide.ad.jp/mawi/	Unlabeled real backbone traffic. Also at CAIDA catalogue.
CAIDA DDoS 2007 CAIDA (2007)	Kumar and Selvakumar (2013), Gulisano et al. (2015), Osanaiye et al. (2016), Hoque et al. (2017), Singh and De (2017), Vidal et al. (2018), Liang and Znati (2019)	https://www.caida.org/catalog/datasets/ddos-20070804_dataset/	1-h anonymised pcap of real DDoS. Also on IEEE DataPort.
UNB-ISCX Lashkari et al. (2017)	Wang et al. (2015), Osanaiye et al. (2016), Jazi et al. (2017), Sharma et al. (2018), Kushwah and Ranga (2021), Gowthul Alam and Michael Raj (2022), Priyadarshini and Barik (2022), Zhou et al. (2023), Ali et al. (2023b)	https://www.unb.ca/cic/datasets/ids.html	Renamed ISCX IDS 2012, now under CIC dataset family.
CIC-IDS2017 Sharafaldin et al. (2018)	Jing et al. (2019), Patil et al. (2019), Prasad et al. (2020a), Prasad et al. (2020b), Çakmakçı et al. (2020), Kasım (2020), Kushwah and Ranga (2021), Gowthul Alam and Michael Raj (2022), Lata and Singh (2022), Agarwal et al. (2022), Zhou et al. (2023), Farhat et al. (2023), Ali et al. (2023a), Ali et al. (2023b), Pandithurai et al. (2024), Binbusayyis (2024), Goyal et al. (2025), Abiramasundari and Ramaswamy (2025), Ijaz et al. (2026)	https://www.unb.ca/cic/datasets/ids-2017.html	Most used (18 studies). Modern attack coverage + realistic traffic.
CSE-CIC-IDS2018 Engelen et al. (2021)	Kanimozhi and Prem Jacob (2019), Thilagam and Aruna (2021), Zhao et al. (2021), Mayuranathan et al. (2022), Abdullayeva (2022), Lata and Singh (2022), Yuan et al. (2023), Zhou et al. (2023), Ali et al. (2023b), Abdullayeva and Suleymanzade (2024), Pandithurai et al. (2024), Binbusayyis (2024), Goyal et al. (2025), Selvy (2025), Abiramasundari and Ramaswamy (2025)	https://www.unb.ca/cic/datasets/ids-2018.html	Also: CIC-IDS2018. Extended CIC-IDS2017 with more attack types.
UNSW-NB15 Moustafa and Slay (2015)	Patil et al. (2019), Kushwah and Ranga (2021), Geetha and Deepa (2022), Gowthul Alam and Michael Raj (2022), Lata and Singh (2022), Mohmand et al. (2022), Zhou et al. (2023), Ali et al. (2023b), Binbusayyis (2024), Rahman et al. (2024), Bellmunt et al. (2025), Ijaz et al. (2026)	https://research.unsw.edu.au/projects/unsw-nb15-dataset	9 attack categories; hybrid real + synthetic UNSW Canberra.
CIC-DDoS2019 Sharafaldin et al. (2019)	Zhou et al. (2023), Pasha et al. (2023), Ali et al. (2023a), Ali et al. (2023b), Najar and Naik (2024), Shaikh et al. (2024), Selvy (2025), Abiramasundari and Ramaswamy (2025), Mandela and Etyang (2025), Mehmood et al. (2025)	https://www.unb.ca/cic/datasets/ddos-2019.html	Reflective DDoS types (SYN, NTP, DNS, etc.).
BoT-IoT Koroniotis et al. (2019)	Geetha and Deepa (2022), Chundru et al. (2022), Gaber et al. (2023), Yuan et al. (2023), Selvan et al. (2023), Sadhwani et al. (2023), Balasubramaniam et al. (2023), Bellmunt et al. (2025)	https://research.unsw.edu.au/projects/bot-iot-dataset	IoT botnet dataset. 72 M + records. UNSW Canberra Cyber Range.
ToN-IoT Moustafa et al. (2018)	Kumar et al. (2021), Gaber et al. (2023), Sadhwani et al. (2023)	https://research.unsw.edu.au/projects/toniot-datasets	IoT/ IIoT telemetry. Also on IEEE DataPort.
InSDN Elsayed et al. (2020)	Alani (2024), Mehmood et al. (2025)	https://ieeexplore.ieee.org/document/9187858	SDN-specific IDS dataset (Elsayed et al., 2020). 343,939 instances.

**Figure 4:**
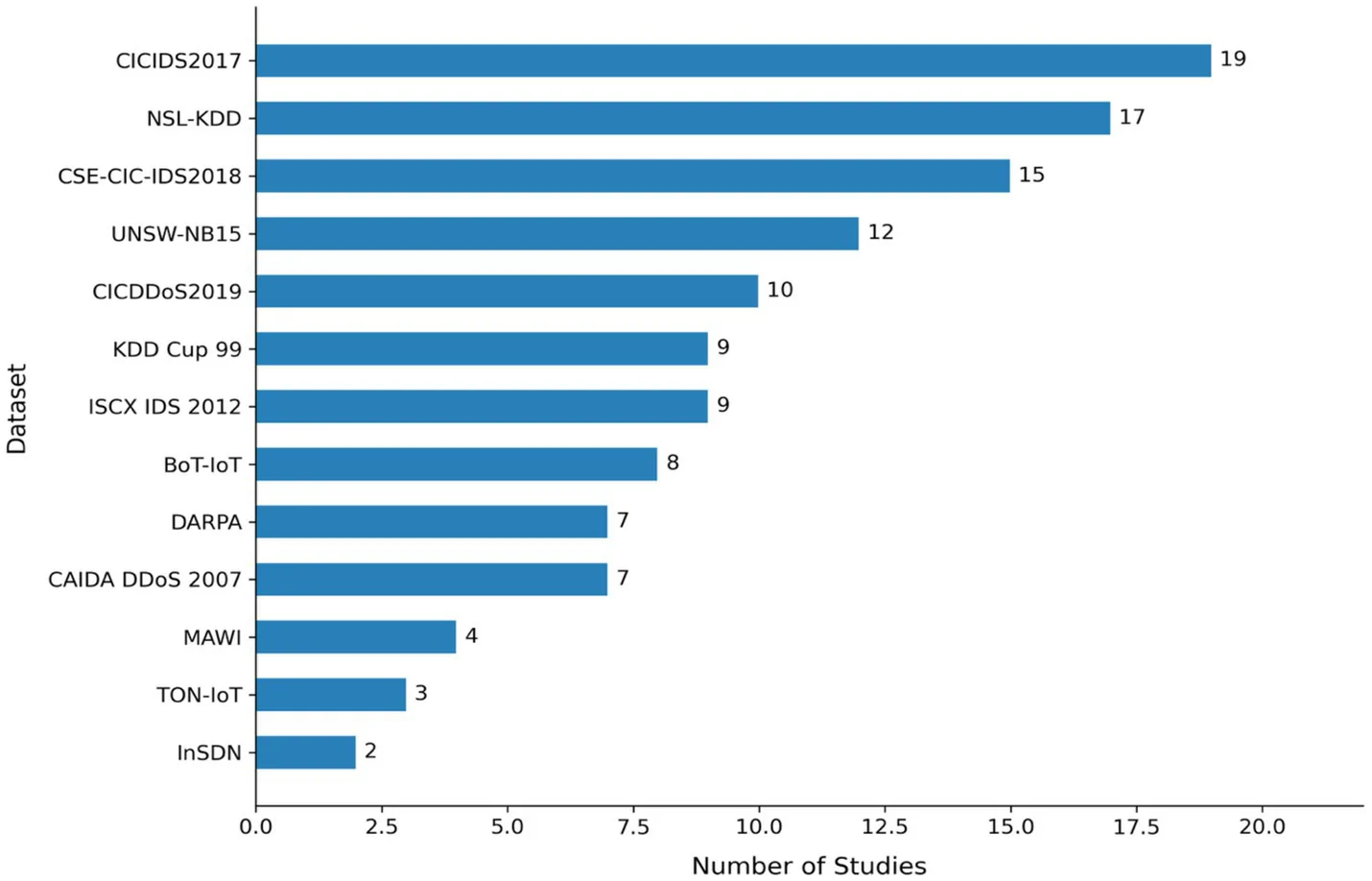
Horizontal bar chart showing the number of studies in the reviewed corpus utilizing each benchmark dataset.

### 6.5 Meta-learning as an adaptive detection paradigm: technical foundations and applicability

The previous sections have made it clear that the most common failure mode of all AI-based DoS and DDoS detection systems is the lack of generalization to the training distribution—whether because of a shift in the distribution, new attack variants, or diverse deployment environments in the cloud—all of which have been established as problems for AI systems. Meta-learning, also known as learning to learn, is about optimizing for performance on a specific task, but also improving the ability to adapt quickly to new tasks, with just a few labeled examples. A meta-trained model is trained with a meta-initialisation, a similarity function or an update rule for fast adaptation to unseen attack types or deployment settings, as opposed to classifiers trained conventionally. Importantly, generalization is not assumed as a property of a large training set, but is an inherent part of the training objective itself.

Episodic N-way K-shot settings are the most common method of evaluating meta-learning, where in each episode, the model is given a K-classes support set of labeled instances, and a query set consisting of one or more instances from N classes to solve. Many such episodes of training develop the model’s ability to generalize over tasks. Ijaz et al. (2026) directly tackle this setting for zero-day intrusion detection, where they show episodic training can help generalize to attack categories not encountered during training.

#### 6.5.1 Algorithm families

There are 3 main families. Optimisation-based methods, such as MAML (Finn et al., 2017) and its first-order variant Reptile, learn initialisations of its parameters that can then be adapted to a new task with only a few gradient steps. In Chen and Ma (2026) MAML is combined with an improved CNN to recognize the attack classes in few-shot DDoS detection using simulated and real-world traffic data, and high recognition accuracy is obtained. This is extended in cloud-fog-edge IoT architectures by Zhang et al. (2025) who develop a MAML-Supervised Contrastive Loss with Learn-to-Forget mechanism on a composite dataset from five major benchmarks. First-order MAML (TayMAML) has been shown by Ju et al. (2026) to yield a better generalization and stability for adaptive task scheduling across heterogeneous edge devices. The Metric-based methods, such as Prototypical Networks (Snell et al., 2017) and Matching Networks (Vinyals et al., 2016) and Relation Networks learn embedding spaces where classes are separated by a distance but do not need fine tuning of the parameters at test time. Ijaz et al. (2026) compare MAML directly with Prototypical Networks, demonstrating that Prototypical Networks are significantly less computationally expensive, and generalize with similar performance, which is favorable for resource-limited edge nodes. The model-based methods, such as Memory-Augmented Neural Networks, are adapted in a forward pass using a differentiable external memory, and are the least-studied family in the context of intrusion detection.

#### 6.5.2 Task formulation

A task may be of DoS/DDoS detection may be a family of attack types (for generalization to new types of attacks), a network or a cloud tenant (for adaptation to a new environment), or a data set (for structured cross-dataset generalization). Each formulation affects the construction of support and query sets. One common problem in both types of formulations is class imbalance, where the traffic from benign flows greatly outnumber the traffic from malicious flows. Ijaz et al. (2026) solve this problem by using the correlation-based feature removal and balancing the episodes, and Zhang et al. (2025) use supervised contrastive loss to push the class embeddings apart in the event of imbalance.

#### 6.5.3 Existing results

The present corpus includes only a single paper that uses transfer learning for intrusion detection (ID) (Farzaneh et al., 2024) show that BiLSTM-based transfer learning yields significant improvement in DDoS detection in 5G environments when labeled data is scarce), but there is a growing number of meta-learning results relevant to ID. This is confirmed by Chen and Ma (2026) and Zhang et al. (2025) who find that MAML-based approaches perform well on the few-shot DDoS detection task on composite multi-benchmark datasets. The most direct comparison is between MAML and Prototypical Networks from Ijaz et al. (2026) which are evaluated on three independent datasets when no training data is available. Bellmunt et al. (2025) prove federated transfer learning to be highly effective for cross-domain knowledge transfer to enhance unknown-attack detection in a data-scarce distributed nodes. Sun et al. (2026) demonstrate the cross domain generalizability of MAML in the context of few-shot log anomaly detection, which is not based on network flow data. Taken together, these results provide proof of concept but not the systematic evaluation on a cloud basis that the present gap analysis identifies as missing with a focus on DoS.

What makes it special, its Meta-learning and not others. Continual learning is designed to retain knowledge instead of rapidly adapting from few examples, and involves the need to have label streams for each new task, which is not possible in adverse conditions where new attack examples are rare. One-class anomaly detection is a very challenging task, as demonstrated in the reviewed literature, and requires no examples of attacks, but results in high false positive rates because of the lack of discriminative supervision. Meta-learning is complementary: When a few labeled examples are given, a meta-trained model can quickly demarcate its decision region in a manner that one-class methods cannot. Self-supervised pre-training decreases the amount of labeled data used but yields fixed representations that are not fine-tuned for fast adaptation. Plain domain adaptation as introduced by Bellmunt et al. (2025) and Farzaneh et al. (2024) is capable of boosting cross domain performance but not to types of attacks not found in the source domain. Meta-learning is a special case of domain adaptation, but also includes the ability to generalize to unseen classes, a more general ability. Three key properties of the case for meta-learning are therefore: explicit optimization for rapid adapting from a small number of labeled examples, generalization to a distribution of tasks over and above a source-target pair, and direct benchmark ability in episodic settings that simulate real-world deployment conditions.

## 7 Comparative analysis of existing studies

The literature reviewed is between 2012 and 2026 with 120 peer-reviewed studies in ten areas of detection. Among them, 88 papers (73.3%) use AI and ML-based detection methods such as SVM, LSTM, CNN, BiLSTM, GRU, DNN, Random Forest, hybrid deep learning, ELM, DBN, XGBoost, neuro-fuzzy, and transfer learning models; 20 papers (16.7%) use non-AI detection methods including statistical detection, entropy-based detection approaches. This distribution indicates the decisive move of the field to the data-driven detection paradigms during the period of the review.

Many of the reviewed studies have accuracy scores near or equal to the maximum on benchmark tests. These statistics should be viewed with some reservation due to several factors. First, in the field of intrusion detection, performances have been shown to be inflated with repetition of the same small datasets, such as NSL-KDD and CIC-IDS2017, by successive studies, which have an advantage of knowing the results of previous studies. Second, inappropriate feature normalization, temporal overlaps and class-aware preprocessing prior to the split of the data leads to data leakage to both the training and test sets, thus inflating reported metrics. Thirdly, publication bias toward positive results implies that studies with high accuracy are more likely to be submitted and accepted for publication, but studies with moderate or negative results are not published, leading to an apparent state of the art that is actually inflated. Fourthly, none of the studies in the corpus report confidence intervals or variance across multiple runs or statistical significance tests, which means that apparent performance differences between model families could not be attributed to actual architectural superiority, as opposed to random variation or dataset specificity. The accuracy data presented in the sections 7.1–7.5 should be interpreted as descriptive data points rather than a ranking, however, and that a lack in statistical reporting itself should be recognized as a methodological gap in the field that requires further investigation.

### 7.1 Detection accuracy progression across paradigms

The accuracy figures across the reviewed studies seem to improve as the studies shift from the earlier days of statistical and threshold-based methods to the more recent deep learning and hybrid methods. The figures are, however, based on studies performed on mixed data sets, class distributions, assessment procedures, and class attack numbers and should be considered a description of observations only, as indicated in Section 7.0. No direct cross-study comparisons of performance are made in this section. All figures quoted here are taken from the original studies and are not ranked or normalized. The initial statistical and unsupervised techniques had set small but baseline performance levels. Casas et al. (2012) reported TPR of 90% and FPR of sub-3.5% with subspace clustering on KDD99, and Wang et al. (2015) 89.30% accuracy caused by probabilistic graphical inference in SDN-cloud settings - the performance limit of pre-learning methods. Xiao et al. (2015) got 96.7% accuracy with correlation-based KNN, Singh and De (2017) got 98.04% with MLP-GA, and Hosseini and Azizi (2019) got 99.7% F-measure with a hybrid Random Forest framework. Deep learning structures also pushed the performance limit, with Kanimozhi and Prem Jacob (2019) scoring 99.97% on CSE-CIC-IDS2018 with hyper-parameter-optimized ANN, Chundru et al. (2022) scoring 99.9% with Decision Tree on BoT-IoT, and Najar and Naik (2024) reporting binary classification accuracy of 99% using CNN in SDN environments–collectively confirming that deep learning consistently outperforms both statistical and classical ML approaches across comparable benchmark conditions.

### 7.2 Model family comparison

The reviewed studies identify five model families that perform and are deployed differently. The tree-based ensemble (RF) models, such as Random Forest (Patil et al., 2019) and Decision Tree (Liang and Znati, 2019), are always 98–99.9 percent compatible with minimal computational cost, and are thus the most deployment-friendly family on IoT, edge, and cloud. Recurrent architectures LSTM (Priyadarshini and Barik, 2022; Deniz and Serttaş, 2023), BiLSTM (Esmaeili et al., 2022; Pandithurai et al., 2024), and hybrid LSTM-GRU (Qureshi et al., 2025) have been shown to perform better at temporal traffic modeling, with 94 to 98.68 percent accuracy in fog, cloud and IIoT environments, Farzaneh et al. (2024) specifically finding that BiLSTM-based transfer learning is better at generalization across attack categories. Deep learning models and optimized classifiers such as SVM-DEHO (Gowthul Alam and Michael Raj, 2022), OCSA-RNN (SaiSindhuTheja and Shyam, 2021), and HBO-BiLSTM (Pandithurai et al., 2024) are also optimized using metaheuristics to identify features that are redundant and model parameters that are optimized despite the significantly higher computational cost. The highest reported accuracies (98.5–99.8%) are achieved by hybrid convolutional-recurrent architectures CNN-LSTM (Issa and Albayrak, 2023), RC-NN (Thilagam and Aruna, 2021), and SVCI-TIPNN (Selvy, 2025) which are able to capture both spatial and temporal dependencies of traffic simultaneously and represent the current state-of-the-art in cloud and SDN detection. The most recent contributions are graph neural networks and transformer-augmented ones: GraphSAGE (Abdullayeva and Suleymanzade, 2024), CNN-Vision Transformer (Shaikh et al., 2024), and DeepDefend CNN-LSTM-Transformer (Ouhssini et al., 2024) with 97.7–99.97 accuracy with multi-flow relational dependencies not visible to traditional feature-vector classifiers.

### 7.3 Dataset usage and evaluation environment comparison

CIC-IDS2017 is the most popular benchmark in the literature reviewed (34 combined CIC-IDS2017/2018 uses), followed by KDD Cup 1999/NSL-KDD (26 uses), CICDDoS2019 and ISCX IDS 2012 (10 and 9 uses respectively) and UNSW-NB15 (12 uses) across multiple studies. Nevertheless, about 34 articles [Of these, 19 studies — (Vissers et al., 2014; Sieklik et al., 2016; Nunes et al., 2017; Cambiaso et al., 2017; Dassouki et al., 2017; Semerci et al., 2018; Kurt et al., 2018; Yadav, 2019; Rethishkumar and Vijayakumar, 2020; Park et al., 2020; Mohammad and Pradhan, 2021; Shah et al., 2021; Sharma and Singh, 2022; Gaber et al., 2023; Hezavehi and Rahmani, 2023; Jayakumari et al., 2024; Shang, 2024; Kumar and Agarwal, 2024; Sharma and Malik, 2025)] — used exclusively synthetic, simulated, or self-generated datasets with no public benchmark for validation. A further 12 studies — (Mitchell and Chen, 2014; Osanaiye et al., 2016; Singh et al., 2016; Iqbal et al., 2016; Coppolino et al., 2017; Khoda Parast et al., 2022; Lata and Singh, 2022; Abdullayeva, 2023; Ali et al., 2023b; Asha and Shanmugapriya, 2024; Abdallah et al., 2024; Afraji et al., 2025) — were survey or review papers that did not involve any experimental evaluation. In three additional studies, Singh and De (2017), Çakmakçı et al. (2020), and Shah et al. (2022) both self-generated or public traffic was used, with the synthetic traffic being the major component in the evaluation environment, such as Nunes et al. (2017), Rethishkumar and Vijayakumar (2020), Virupakshar et al. (2020), Hezavehi and Rahmani (2023), used only synthetically generated or simulation-based data with no comparison to real-life operational traffic (the most consistent evaluation limitation in the corpus of reviewed articles). Research that was assessed on several datasets, including (Gowthul Alam and Michael Raj, 2022) on NSL-KDD, UNSW-NB15, ISCX, and CIC-IDS2017, and Geetha and Deepa (2022) on UNSW-NB15, NSL-KDD99, BoT-IoT, and TUIDS, showed more robust and generalizable performance claims than single-dataset studies, reinforcing the importance of multi-benchmark evaluation for credible detection research.

### 7.4 Parallel model implementation across studies

Several detection architectures were simulated separately in several studies, allowing direct comparative observations. Priyadarshini and Barik (2022) (mog-SDN environment 97.77% detection) and Deniz and Serttaş (2023) (OpenStack cloud 98% detection) and Agarwal et al. (2021) (FRC attack detection 0.0% FPR, this figure reflects rounding to one decimal place rather than a literally zero false positive rate) independently investigate LSTM-based detection in cloud and fog environments, respectively. Adi et al. (2017), Kasım (2020), Ali et al. (2023a) independently applied SVM: stealthy detection of HTTP/2-based applications (0% false alarms), stealthy detection of AE-SVM-based applications (99.41% accuracy), and stealthy detection of SDN DDoSes (95.57% accuracy)-showing that SVM achieved competitive performance across multiple studies. Random Forest was utilized in six independent studies (Hosseini and Azizi, 2019; Patil et al., 2019; Mohmand et al., 2022; Gaber et al., 2023; Sadhwani et al., 2023; Abiramasundari and Ramaswamy, 2025), with a consistent accuracy of 96–99.9 percent in the cloud, IoT, and network setting, thus making it the most validated classical ML model in the corpus. Semerci et al. (2018) (Mahalanobis distance-based DCPM, F-score 0.88) and Kurt et al. (2018) (Bayesian multiple change-point model, F-score 0.94) independently applied change-point models (Bayesian and probabilistic change-point models) to SIP-based DDoS detection, both without labeled data on the identical threat, with the Bayesian approach demonstrating superior detection precision.

### 7.5 Detection domain and deployment scope comparison

The most significant research area is cloud-based detection with 30 studies, which demonstrates the rising prominence of cloud infrastructure in terms of enterprise functionality and attack areas. The best binary classification accuracy ceiling (99.95–99.99%) was SDN-based detection because of programmable control-plane visibility, although was limited by reliance on centralized controllers and specialized labeled datasets. IoT and edge detectors, with 14 studies (Sharma et al., 2018; Kumar et al., 2021; Zhao et al., 2021; Sambangi et al., 2022; Reddy and Shyam, 2022; Esmaeili et al., 2022; Priyadarshini and Barik, 2022; Gaber et al., 2023; Yang et al., 2023; Lai et al., 2023; Sadhwani et al., 2023; Alani, 2024; Binbusayyis, 2024; Qureshi et al., 2025), had the special challenge of considering accuracy versus resource constraints - with Sadhwani et al. (2023) reporting 100% accuracy with lightweight feature-selected models and the initial performance results of 12 studies (Casas et al., 2012; García et al., 2014; Gulisano et al., 2015; Hoque et al., 2017; Jazi et al., 2017; Vidal et al., 2018; Jing et al., 2019; David and Thomas, 2019; Prasad et al., 2020b; Çakmakçı et al., 2020; Bouyeddou et al., 2020; Rahman et al., 2024; Wang et al., 2025) set the baseline of performance on which all future ML and DL models are implicitly compared, and the 90% TPR result (Casas et al., 2012) to the 99.97% accuracy result (Ouhssini et al., 2024) over a thirteen-years quantifying the cumulative impact of the field’s transition toward adaptive deep learning frameworks.

### 7.6 Systemic evaluation practice gaps

A number of evaluation practice gaps are identified across all sections 7.1 to 7.5 and warrant discussion together. First, the false negative rate is seldom reported and is directly related to missed attack detection in operational deployments where the cost of an intrusion is much higher than the cost of a false alarm (since the overwhelming majority of studies report accuracy, precision, recall and F1-score – leaving out the FNR). Second, detection latency is rarely included in any of the reported metrics, including those studies that make a specific effort to show detection in real time, so it is unclear whether the proposed frameworks would be able to detect attacks within the sub-second timeframe of live cloud environments. Third, many studies published since 2018 are purely based on the CICFlowMeter feature extraction tool, which can lead to a systematic tool dependency that could overestimate the comparability of the studies: features extracted by different versions of CICFlowMeter are not necessarily the same, and studies that are run with alternative feature extraction tools are not necessarily comparable, even if the same dataset is used. Fourth, the lack of consistency in method of reporting metrics across studies (some studies report macro-averaged metrics, others report weighted or per-class metrics without specifying which) makes it difficult to make meaningful comparisons within the same study. All four of these gaps point toward the need for standardized testing protocols that contain the following as required reporting: FNR, Detection latency, Tool version reporting and explicit metric averaging strategy.

### 7.7 Key comparative observations

This analysis brings out three cross-cutting observations. First, detection accuracy is not a sufficient comparative measure, as studies with near-perfect accuracy on single synthetic datasets like (Rethishkumar and Vijayakumar, 2020) at CloudSim and Virupakshar et al. (2020) at self-generated OpenStack traffic are not directly comparable to studies with near-perfect accuracy on multiple public benchmarks like (Patil et al., 2019) at the cost of increased computational overhead. Second, the main trade-off that has not yet been addressed is computational efficiency - even the most accurate architectures such as Goyal et al. (2025), Selvy (2025), Binbusayyis (2024) explicitly state that their architectures have prohibitive computational power making them unsuitable for use in resource-constrained IoT and edge applications. Third, the lack of a common evaluation framework - which is emphasized by survey contributions of García et al. (2014), Ali et al. (2023b), Abdallah et al. (2024), and Afraji et al. (2025) implies that performance comparisons across studies is highly contextual and no particular model can be unambiguously determined as best in all areas of detection, data sets, and deployment contexts–a gap that future meta-learning frameworks, by virtue of their rapid cross-domain adaptability, are uniquely positioned to address.

## 8 Research challenges

Although there have been major improvements in the fields of DoS and DDoS detection within the statistical, machine learning, and deep learning paradigms, several unresolved issues continue to stand in the way of effective and deployable intrusion detection systems. First issue in the reviewed literature is the over-reliance on benchmark datasets, particularly CIC-IDS2017, NSL-KDD and KDD99, which fail to reflect the traffic and protocol heterogeneity, as well as the dynamic nature of attacks in real-life network setting. Studies such as Casas et al. (2012), Mohmand et al. (2022), SaiSindhuTheja and Shyam (2021) also showed that the imbalance in the classes in these datasets has a systematic effect of biasing the detection models with majority attack classes, and the lack of standardized evaluation measures across studies [identified by García et al. (2014) and Liang and Znati (2019)]-limits meaningful cross-study comparison and reproducibility.

The second challenge is related to the accuracy of the offline experiments and the possible implementation in real-world scenarios. The reviewed frameworks, such as those by Virupakshar et al. (2020), Rethishkumar and Vijayakumar (2020), Hezavehi and Rahmani (2023), and Nunes et al. (2017) were only evaluated in a simulated or synthetically generated network and not on actual networks. Meanwhile, architectures that are more computationally demanding like ones of Goyal et al. (2025), Ouhssini et al. (2024), Selvy (2025) have been proposed which consume resources that are not readily available in the resource-constrained contexts of Zhao et al. (2021), Lai et al. (2023), and Sadhwani et al. (2023) respectively.

A third challenge that remains elusive is the detection of a zero-day attack and generalization. The deep learning models of Esmaeili et al. (2022) and Deniz and Serttaş (2023) as well as Agarwal et al. (2022) trained on pre-defined benchmark distributions suffer significantly if faced with new or unknown types of attacks. While the transfer learning method of Farzaneh et al. (2024) partially solves the problem of cross-domain generalization, it is claimed that domain shift between the source and target network environments is still a reported problem. Furthermore, the literature examined in this paper highlights a key and unanswered question: whether there are any frameworks that support meta-learning to rapidly adapt to a heterogeneous network environment using only a small number of labeled samples, which is also partially suggested by the label-free detection methods of Abdullayeva (2022) and Çakmakçı et al. (2020).

While meta-learning presents a compelling paradigm for adaptive DoS and DDoS detection in cloud environments, its inherent limitations must be acknowledged. First, the meta-training phase incurs substantially higher computational cost compared to conventional supervised learning, as bi-level optimization over a distribution of tasks is significantly more expensive than standard empirical risk minimization-a constraint that is particularly acute for resource-limited cloud edge deployments. Second, meta-learning frameworks are sensitive to the quality and diversity of the task distribution used during training; when sampled tasks do not adequately represent the target attack distribution, negative transfer can occur, where prior meta-knowledge actively degrades performance on new detection tasks rather than improving it, as confirmed by Ijaz et al. (2026) who found MAML training cost substantially higher than Prototypical Networks across all evaluated datasets. Third, in continual or lifelong learning scenarios where new attack types are introduced incrementally, meta-learned models remain susceptible to catastrophic forgetting-a critical concern in adversarial network environments where attack diversity grows continuously. Fourth, the cybersecurity domain suffers from a severe scarcity of publicly available meta-learning benchmarks specifically designed for intrusion detection; most existing datasets were constructed for supervised classification rather than few-shot or cross-domain evaluation., which limits rigorous assessment of meta-learning-based detection frameworks. Addressing these challenges-through task-aware sampling strategies, memory-augmented architectures, computationally efficient meta-optimizers such as TayMAML (Ju et al., 2026), and the construction of purpose-built cybersecurity meta-learning benchmarks-represents a critical priority for future research.

The reviewed literature still does not adequately reflect the issues of privacy, explainability and trust. Somani et al. (2016), Aldribi et al. (2020), and Patil et al. (2019) analyzed multi-tenant cloud configurations and found that there is a tradeoff between tenant data visibility and detection effectiveness, however, none of the reviewed studied tenets had federated or privacy-preserving detection engines. Moreover, the black-box nature of high performing deep learning models like DA-Dense-BiGRU (Goyal et al., 2025), GraphSAGE (Abdullayeva and Suleymanzade, 2024) and DeepDefend (Ouhssini et al., 2024) presents a clear challenge to deployment in regulated and mission-critical network contexts where technical decisions must be interpretable. The work of Abdallah et al. (2024) on explainability, Afraji et al. (2025) on scalability, and Asha and Shanmugapriya (2024) on integrated assessment framework all validate the need to address explainability, scalability, and integrated assessment frameworks as the most pressing unmet needs in this field.

This review was not registered in advance in PROSPERO or similar register before the beginning of the review process and this constitutes a methodological limitation. No dual independent screening was carried out, nor were inter-rater agreement statistics calculated as there was only a single reviewer (the first author) carrying out screening and supervised by a supervisor. Furthermore, the review is limited to articles that were published in English, so some relevant contributions from the non-English speaking research communities may have been missed. Furthermore, a supplemental search was carried out using specific terms related to meta-learning and few-shot learning during the revision process that reveals six more papers (115–120) were added to the corpus, which ultimately includes 120 papers.

## 9 Future research directions

In this review, we identified several gaps, with the lack of meta-learning frameworks for rapid low-label adaptation being the most significant gap for next-generation cloud-based intrusion detection. Another direction for future research is meta-learning systems that can learn effectively from a small number of labeled samples, rather than requiring vast retraining in order to adapt to attack patterns that have not been encountered before and heterogeneous network environments, as demonstrated by Farzaneh et al. (2024). The model compression methods that are closely related to the aforementioned, but simpler and more lightweight, such as pruning, quantization, and knowledge distillation, would help bridge the gap between the complex architectures of Goyal et al. (2025) and Selvy (2025) and the resource constrained edge and fog networks studied by Zhao et al. (2021), Lai et al. (2023), and Sadhwani et al. (2023) and enable meta-learning-driven detection in real-life resource-limited deployments.

Trust, explainability, and privacy is another top priority direction. While multi-tenant cloud systems studied by Somani et al. (2016), Aldribi et al. (2020), and Patil et al. (2019) show that there is a natural conflict between tenant data visibility and detection effectiveness, none of the systems studied in these three papers use federated or privacy-preserving methods. In future, federated learning can be combined with differential privacy and homomorphic encryption, which can be adapted to the decentralized trust principles proposed by Kotel et al. (2023) and Jayakumari et al. (2024) and allow a joint detection without losing the confidentiality. Meanwhile, high-accuracy architectures such as DA-Dense-BiGRU (Goyal et al., 2025), GraphSAGE (Abdullayeva and Suleymanzade, 2024) and DeepDefend (Ouhssini et al., 2024) remain black-box models, which is not a major concern in an unregulated space, but poses a significant hurdle when it comes to regulated environments. It is therefore crucial to introduce explainable AI (XAI) mechanisms like SHAP, LIME and attention-based visualization into these architectures as discussed by Abdallah et al. (2024), Afraji et al. (2025), and Asha and Shanmugapriya (2024).

In addition to these two priorities, realistic benchmark datasets that represent encrypted traffic, SDN flows, slicing in 5G, and IIoT patterns are essential to break away from the dataset stagnation pointed out by García et al. (2014) and Ali et al. (2023b) and to enable reproducible detection research. Lastly, the current architectural level of next generation intrusion detection, in which metrics from temporal graph neural networks, vision transformers, and multi-modal detection systems combine to provide the most powerful form of threat detection – including honeypot telemetry and NLP-driven threat intelligence, as introduced by Alani (2024) and Sharma and Malik (2025) – is most likely to be supported by meta-learning driven adaptability.

## 10 Conclusion

In this review, we systematically followed the development of DoS and DDoS attack detection techniques, ranging from statistical and anomaly-based detection to more complex domain-aware, machine learning, and deep learning detection in cloud, SDN, IoT, edge, and hybrid network environments. As shown in the literature review, detection accuracy has improved significantly in the last decade; under controlled experimental conditions, state-of-the-art deep learning architectures are capable of classifying with near-perfect accuracy.

But the most difficult and unanswered question in the field is getting from experimental accuracy to real-world deploy ability. The use of a small set of benchmark datasets, class imbalance, limited ability to generalize to novel attack types, expensive compute requirements in resource limited environments, and the continued lack of explainability and privacy-preserving approaches all limit the ability to use even the most sophisticated of detection systems in real world cloud and distributed network deployments.

This background makes the most intriguing and practical approach for future intrusion detection research in the cloud environment: meta-learning. Meta-learning can directly solve the problems of the zero-day detection gap, cross domain generalization problems and label scarcity, which existing transfer learning and deep learning methods have not been fully solved. This review signals a key research path that combines the principles of meta-learning with federated, privacy-preserving approaches and explainable AI—a path that is both principled and practically relevant and is poised to be the most critical and impactful next steps toward truly adaptive, trustworthy, and deployable intrusion detection systems in modern network infrastructures.

## Data Availability

The original contributions presented in the study are included in the article/Supplementary material, further inquiries can be directed to the corresponding author.

## Glossary

5G: Fifth Generation
AE: Autoencoder
AIS: Artificial Immune System
ALO: Ant Lion Optimization
ANN: Artificial Neural Network
API: Application Programming Interface
ARP: Address Resolution Protocol
AWS: Amazon Web Services
BBA: Binary Bat Algorithm
BBOA: Brown Bear Optimization Algorithm
BDI: Belief-Desire-Intention
BiGRU: Bidirectional Gated Recurrent Unit
BiLSTM: Bidirectional Long Short-Term Memory
BoT: Botnet of Things
BRS: Balanced Random Sampling
CAIDA: Center for Applied Internet Data Analysis
CIC: Canadian Institute of Cybersecurity
CNN: Convolutional Neural Network
CPU: Central Processing Unit
CRDO: Chaotic Red Deer Optimization
CRPS: Continuous Ranked Probability Score
CRT: Chinese Remainder Theorem
CSP: Cloud Service Provider
CUSUM: Cumulative Sum Control Chart
DARPA: Defense Advanced Research Project Agency
DBSCAN: Density-Based Spatial Clustering of Application with Noise
DCNN: Deep Convolutional Neural Network
DCPM: Detection Change Point Model
DBN: Deep Belief Network
DDoS: Distributed Denial of Service
DEHO: Discrete Elephant Herding Optimization
DL: Deep Learning
DKNN: Deep Kronecker Neural Network
DNN: Deep Neural Network
DNS: Domain Name System
DoS: Denial of Service
DRL: Deep Reinforcement Learning
DSA: Deep Stacked Autoencoder
DT: Decision Tree
DTL: Deep Transfer Learning
ECP: E-Divisive Change Point
ECDSA: Elliptic Curve Digital Signature Algorithm
EDoS: Economic Denial of Sustainability
ELM: Extreme Learning Machine
ES: Exponential Smoothing
FPGA: Field-Programmable Gate Array
FPR: False Positive Rate
FRC: Fraudulent Resource Consumption
FTP: File Transfer Protocol
GA: Genetic Algorithm
GAN: Generative Adversarial Network
GNN: Graph Neural Network
GRU: Gated Recurrent Unit
GWO: Grey Wolf Optimization
HBO: Honey Badger Optimization
HTTP: Hypertext Transfer Protocol
IaaS: Infrastructure as a Service
ICMP: Internet Control Message Protocol
IDS: Intrusion Detection System
IDPS: Intrusion Detection and Prevention System
IIoT: Industrial Internet of Things
IoMT: Internet of Medical Things
IoT: Internet of Things
ISCX: Information Security Centre of Excellence
KDD: Knowledge Discovery in Databases
KPCA: Kernel Principal Component Analysis
LAN: Local Area Network
LSTM: Long Short-Term Memory
MANET: Mobile Ad-hoc Network
MCC: Mathews Correlation Coefficient
ML: Machine Learning
MLP: Multi-Layer Perceptron
MPA: Multi-class Passive-Aggressive
MQTT: Message Queuing Telemetry Transport
NaHiD: Network-aware Host-based IDS
NIDS: Network Intrusion Detection System
NLP: Natural Language Processing
NSL-KDD: Network Security Lab KDD
OBS: Optical Burst Switching
OCSA: Oppositional Crow Search Algorithm
OpenStack: Open-Source Cloud Platform
PaaS: Platform as a Service
PCA: Principal Component Analysis
PBFT: Practical Byzantine Fault Tolerance
QoS: Quality of Service
RBM: Restricted Boltzmann Machine
RNN: Recurrent Neural Network
RAD: Runtime Anomaly Detection
RF: Random Forest
RFE: Recursive Feature Elimination
SaaS: Software as a Service
SDN: Software-Defined Networking
SIP: Session Initiation Protocol
SOM: Self-Organizing Map
SVM: Support Vector Machine
SVD: Singular Value Decomposition
TCP: Transmission Control Protocol
TFTP: Trivial File Transfer Protocol
TPR: True Positive Rate
TNR: True Negative Rate
TTL: Time to Live
TUIDS: Tezpur University IDS
UDP: User Datagram Protocol
UFS: Unsupervised Feature Selection
UNSW: University of New South Wales
VANET: Vehicular Ad-hoc Network
VM: Virtual Machine
VMI: Virtual Machine Introspection
VoIP: Voice Over Internet Protocol
VPN: Virtual Private Network
VSI-DDoS: Very Short Intermittent DDoS
WGAN: Wasserstein Generative Adversarial Network
WAN: Wide Area Network
WSN: Wireless Sensor Network
XAI: Explainable Artificial Intelligence
XGBoost: Extreme Gradient Boosting
