# Supplementary material for: A systematic review of DoS and DDoS attack detection in cloud environments: evolutionary trends and the emerging role of meta-learning for cross-domain adaptability
Source: Front Artif Intell. 2026 Oct 21;9:1958096. doi: 10.3389/frai.2026.1958096 (PMC13731317; doi:10.3389/frai.2026.1958096)
Supplement: Supplementary file 2 [file Table_2.DOCX]

| **Reference** | **Model** | **Study Area** | **Dataset** | **Feature selection method** | **Feature extraction method** | **Weakness/ Limitations** | **Finding** | **Result** |
| --- | --- | --- | --- | --- | --- | --- | --- | --- |
| Casas et al. 2012 [1] | UNIDS(Unsupervised Network Intrusion Detection System) using Sub-Space Clustering+DBSCAN+EA4RO | Network Intrusion Detection System | KDD99, MAWI, METROSEC | **NA** | Traffic Features | High Computational cost for large traffic | Detects Unknown attacks without labeled data | TPR 90%, FPR<3.5% |
| Arshad et al. 2013 [2] | Decision Tree based intrusion Severity Analysis | Cloud Intrusion Detection | Programmatically generated dataset | Dynamic weighted prioritization of security requirements | System call-based security features | Large Decision tree may increase computational overhead | ML can effectively analyze intrusion severity using VM-specific parameters | Enables automated intrusion severity analysis in cloud environments |
| Stevanovic et al. 2013 [3] | Self-Organizing Map (SOM) +Modified Adaptive Resonance Theory 2(MART2) | Web Server log-based intrusion detection | York University web server log dataset | Statistical feature analysis using significance test | Web session behavioral feature from server logs | Crawlers mimic human behavior | Behavioral patterns differentiate users | Unsupervised clustering detects malicious crawlers |
| Raj Kumar et al. 2013 [4] | NFBoost (ANFIS ensemble) | Network/DDoS detection | KDD99,CAIDA,Conficker,UNINA | Information Gain | Network traffic features | High Computational Complexity | Ensemble neuro-fuzzy improves detection | Effective DDoS detection with reduced false alarms |
| Vissers et al. 2014 [5] | Gaussian-based outlier detection defense system | Cloud web services security | Logged SOAP/HTTP request dataset | Statistical feature filtering | SOAP/XML features | Requires training dataset of normal traffic | Outlier detection can identify SOAP/HTTP requests | Mitigates application-layer DoS |
| Garcia et al. 2014 [6] | BClus, CAMPNEP, BotHunter | Botnet detection | CTU-13 Botnet Dataset(NetFlow-based real botnet traffic) | Flow aggregation and clustering rules | NetFlow traffic features | Limited botnet diversity | Comparison framework helps evaluate detection methods | Benchmark comparison for botnet detectors |
| Mitchell et al. 2014 [7] | Survey of IDS techniques | Wireless network intrusion detection | NA | NA | NA | NA | No experimental evaluation | Identifies gaps in wireless IDS research |
| Wang et al. 2015 [8] | STONE (Streaming-based Online Network defense Engine) | Large-scale network/DDoS detection | CAIDA 2007, OptoSUNET | Traffic aggregation | IP prefix and traffic flow features | Limited to flooding attacks | Streaming analytics improves real-time detection | Scalable and accurate DDoS mitigation |
| Xiao et al. 2015 [9] | CKNN(Correlation-based KNN) | Data center network security | MAWI,KDD99,Real data center traffic | Correlation-based Feature Selection | Flow-based statistical traffic features | Depends on labeled data | Correlation improves detection | Better detection accuracy and lower overhead than traditional KNN |
| Wang et al. 2015 [10] | DaMask(Graphical probabilistic model+ SDN architecture) | Cloud computing and SDN-based enterprise networks | UNB-ISCX Intrusion Detection Dataset | Chow-Liu algorithm for automatic feature selection | Flow-based network traffic feature from packet/flow statistics | Higher overhead in SDN communication | SDN architecture improves DDoS detection and mitigation flexibility | Graphical model with SDN enables effective adaptive DDoS protection |
| Osanaiye et al. 2016 [11] | Change-point detection framework | Cloud DDoS defense | UNB-ISCX, CAIDA, DARPA,KDD99 (reviewed datasets) | Packet inter-arrival time | Flow-based traffic features | Lack of updated labeled dataset | IAT helps detect abnormal traffic | Conceptual framework for cloud DDoS detection |
| Somani et al. 2016 [12] | Cloud resource allocation system model | Cloud Computing DDoS/EDoS impact analysis | PlanetLab workload traces | Resource utilization metrics | VM workload and traffic behaavior | Focuses on effect analysis rather than detection method | DDoS attacks impact non-target VMs and cloud resources | Demonstrates collateral damage in multi-tenant cloud systems |
| Singh et al. 2016 [13] | 3-tier cloud security | Cloud computing security issues and threats | NA | NA | NA | No experimental validation | Highlights key cloud security threats | Provides conceptual framework for improving cloud security |
| Iqbal et al. 2016 [14] | Cloud attack taxonomy | Cloud computing security and intrusion detection | NA | NA | NA | No real dataset evaluation | Classifies cloud attacks and IDS deployment | Improves understanding of cloud attack detection strategies |
| Sieklik et al. 2016 [15] | TFTP amplification attack evaluation model | DDoS amplification attack | Simulated TFTP traffic dataset | NA | Network traffic metrics | Only analyzes TFTP-based amplification attack | TFTP can amplify DDoS traffic significantly | Demonstrates vulnerability of TFTP to DDoS amplification |
| Coppolino et al. 2017 [16] | Multi-layer cloud security analysis framework | Cloud Computing security | NA | NA | NA | No quantitative evaluation | Multi-layer security is required to handle evolving cloud threats | Comprehensive mapping of threats and defense mechanisms across cloud layers |
| Nunes et al. 2017 [17] | BDI-based multi-agent system | Network resilience & DDoS mitigation | Simulated dataset using PReSET+ OMNeT++ | NA | Traffic behavior & anomaly detection features | Complex multi-agent coordination and lack of real world validation | BDI agents provide flexible and adaptive DDoS mitigation | Outperforms ECA with better adaptability and robustness |
| Adi et al. 2017 [18] | Stealthy HTTP/2 DoS model with ML classifiers(Naïve Bayes, Decision Tree, JRip, SVM) | HTTP/2 network security & web services | Synthetic HTTP/2 dataset (DOBBS + simulated traffic) | Information Gain, Gain Ratio | Statistical traffic features | Lack of real HTTP/2 dataset; relies on simulated traffic | Stealthy attacks evade ML detection | ML detects attacks but higher false alarms for stealthy traffic |
| Cambiaso et al. 2017 [19] | SlowComm(Slow DoS attack model) | Application-layer DoS in internet services | Experimental testbed(Apache server, FTP, SMTP) | NA | Network traffic behavior & connection features | Hard to detect but mitigation still possible using modules like mod_security | Low-bandwidth, protocol-independent DoS attack | Efficient, low-resource attack that can sustain DoS with minimal bandwidth |
| Hoque et al. 2017 [20] | NaHiD(Correlation-based + FPGA) | Real-time DDoS detection in network/cloud | CAIDA 2007, DARPA, TUIDS | Manual minimal feature subset (3 features) | Entropy of source IP, variation index of source IP, Packet rate | High dependency on threshold & limited to binary classification | Efficient Correlation measure for real-time detection | Achieves high accuracy with ultra-fast hardware-based detection |
| Dassouki et al. 2017 [21] | Temporal + Fingerprint-based Detection & Whitelist Mitigation | SIP-based VoIP security( Cloud Environment) | Real-time simulated cloud-based SIP traffic (AWS & DigitalOcean setup) | Protocol-based feature selection | Temporal features + SIP message fingerprinting | Cannot handle new unseen legitimate users during attack | Combining temporal behavior with SIP improves DDoS detection | Provides fast detection, low false alarms, and efficient resource usage |
| Singh et al. 2017 [22] | MLP-GA | Application-layer DDoS detection(Web servers) | EPA-HTTP, CAIDA 2007, Slowloris | Manual selection of 4 traffic features | Traffic behavior-based features | Limited to selected features and difficulty distinguished flash traffic vs attacks | Hybrid MLP-GA improves detection over traditional classifiers | Effective and robust detection of application-layer DDoS with improved accuracy and low false positives |
| Jazi et al. 2017 [23] | Nonparametric CUSUM-based detection | Application-layer DoS (HTTP attack in web servers | ISCX dataset +web server traffic + generated attack traces | Heuristic feature selection | Traffic behavior features | Sampling distorts traffic & reduces detection accuracy | CUSUM effectively detects both high and low-rate attacks | Accurate and robust detection under real-world conditions |
| Hasan et al. 2018 [24] | DCNN (Deep Convolutional Neural Network) | BHP flooding attack detection in Optical burst Switching networks | BHP Flooding Attack Dataset (UCI Repository) | No explicit selection | Automatic feature learning via CNN | Small dataset size limits generalization | DCNN outperforms traditional ML models (SVM, KNN, NB) | Highly accurate and robust detection |
| Semerci et al. 2018 [25] | DCPM (Adaptive Mahalanobis Distance + Spectral Clustering) | SIP/VoIP network DDoS detection | Simulated SIP traffic (Asterisk server + NOVA V-Spy) | NA | Statistical features | Limited real-world validation | Effective real-time detection without labeled data | Robust and adaptive anomaly detection |
| Vidal et al. 2018 [26] | Adaptive Artificial Immune System with entropy prediction | DoS/DDoS flooding detection in IP networks | KDD’99, CAIDA, CAIDA’08, UCM real traffic, DDoSIM generated data | NA | Entropy-based traffic features (Renyi entropy + Holt-Winters) | Limited handling of advanced evasion attacks | AIS enables adaptive and distributed detection | Effective real-time detection with low false alarms |
| Kurt et al. 2018 [27] | Bayesian Multiple Change Point Model (Hidden Markov-based) | SIP-based VoIP DDoS detection | Simulated SIP network data (Boun-Sim + Nova V-Spy attacks) | NA | SIP message counts + server resource metrics | Limited real-world validation | Bayesian framework effectively detects abrupt traffic changes | High detection accuracy( ~0.94 F-score with smoothing) |
| Sharma et al. 2018 [28] | Deep Belief Network(DBN) + SDN-based OpCloudSec architecture | Cloud-SDN-IoT network security (DDoS detection) | UNB-ISCX dataset | NA | Deep hierarchical feature learning | Simulation-based SDN environment, limited real-world validation | DBN-based model improves adaptive DDoS detection in IoT-cloud | High detection accuracy and better precision/recall than SVM, NB, SOM |
| Liang et al. 2019 [29] | Decision Tree + feature-based ML comparison | Internet backbone DDoS detection | CAIDA + DARPA dataset | Information Gain/ Gini Index | Packet header-based traffic features | Performance varies across traffic types and imbalance issues | Feature selection significantly impacts detection accuracy | High accuracy but no single model is consistently best |
| Jing et al. 2019 [30] | CRT-based reversible Sketch + MM-CUSUM (Hybrid: Statistical +Data Structure) | Network level DDoS flooding detection | MAWI dataset + CIC-IDS2017 | Threshold-based (τ, δ) + EWMA-based statistical filtering | Multi-dimensional traffic features(TCP, UDP, ICMP counts) | Sensitive parameter tuning (τ, δ, time interval) and requires careful configuration | Reversible sketch enables efficient identification of attack sources with adaptive detection | High TPR(~98-100%) and low FPR(~0.6-1.35%) with low computational complexity |
| David et al. 2019 [31] | Dynamic Thresholding (Statistical) | Flow-based network DDoS detection | DARPA 98, DARPA 2000, University Generated dataset | Threshold-based | Packet header features(IP, protocol, packet count) | Sensitive to parameter tuning and traffic variation | Dynamic threshold improves detection over static methods | High detection accuracy with low computation time |
| Yadav et al. 2019 [32] | WFCM-AANN (Hybrid ML) | Cloud-based malware detection (IDS) | Simulated cloud dataset(CloudSim + generated data) | Weighted clustering-based (feature importance W) | Traffic attributes & behavioral features (packet frequency, attributes) | High false alarm rate and complexity in feature learning | Hybrid WFCM + AANN improves malware detection | Better precision and recall than FCM-SVM & FCM-NN |
| Hosseini et al. 2019 [33] | Hybrid ML ( Ensemble + Incremental learning) | Network-based DDoS detection (client-proxy architecture) | NSL-KDD + Alkasassbeh dataset | Forward feature selection | Traffic features + statistical attributes | Higher system complexity & dependency on multiple classifiers | Hybrid multi-algorithm framework improves detection & handles new attacks | Random Forest performs best with high accuracy and improved detection |
| Kanimozhi et al. 2019 [34] | ANN (MLP with Hyperparameter Tuning) | Network intrusion detection (Botnet attack classification) | CSE-CIC-IDS2018 | Hyperparameter tuning (GridSearchCV) | CICFlowMeter extracted 80 traffic features | High computational cost & overfitting risk | Optimized ANN significantly improves IDS performance | Very high accuracy and strong classification performance |
| Patil et al. 2019 [35] | Random Forest + Binary Bat Algorithm (Hybrid) | Cloud network intrusion detection | UNSW-NB15, CICIDS-2017 | Extended Binary Bat Algorithm (FSFF + CAFF) | 26 optimized traffic features (flow-based) | High complexity & dependency on feature optimization | Optimizes features significantly improve detection accuracy | >99% accuracy with low false positive rate |
| Aldribi et al. 2020 [36] | Multivariate Change Detection (E-Div + Gradient Descent) | Hypervisor-level cloud intrusion detection | ISOT-CID (custom cloud dataset) | NA | Instance-oriented feature model | Limited dataset size & scalability issues | Hypervisor-level behavioral analysis improves detection of unknown attacks | High detection capability with better anomaly detection than signature IDS |
| Garg et al. 2020 [37] | En-ABC (RBM+ UKF + ABC-FCM) | Cloud anomaly detection | NSL-KDD, NAB, IBRL + synthetic cloud data | RBM- based feature selection | Deep feature learning (RBM) + clustering (FCM) | High computational complexity due to multi-stage processing | Hybrid evolutionary + deep learning improves anomaly detection accuracy | Effective detection of point & collective anomalies |
| Prasad et al. 2020 [38] | Bayesian + Rough Set Hybrid (BRS) | Network Intrusion Detection | CICIDS2017 | Rough Set + Probability-based feature ranking | Probabilistic feature learning (FPE) | Manual preprocessing and difficulty in selecting optimal feature subset | Hybrid Bayesian- Rough approach improves detection accuracy | High accuracy with reduced complexity using selected features |
| Prasad et al. 2020 [39] | UFS + CCI-based Clustering | Network IDS (LAN + MANET) | KDD’99, CICIDS2017, Wormhole | Unsupervised Feature Selection (UFS) | Distance-based clustering features (micro-clusters with centroid initialization) | Manual preprocessing and higher time/space complexity | UFS + cluster initialization improves clustering accuracy and reduces iterations | Achieves high DR and accuracy with reduced complexity |
| Rethiskumar et al. 2020 [40] | State Transition Model (STM) + Markov Chain | Cloud (VM-level DoS attack detection) | CloudSim (simulated) | Risk metric-based feature evaluation | State-based representation of VM behavior (& VM states using Markov transitions) | Only simulated environment; lacks real dataset validation | STM dynamically tracks VM risk states for attack detection | Improves detection accuracy and increases attacker cost significantly |
| Park et al. 2020 [41] | Experimental evaluation of VSI-DDoS(Low-rate pulsing attack) | Cloud-based web server (RUBBoS 3-tier architecture) | RUBBoS benchmark + synthetic traffic ( bot + legitimate users) | NA | Network traffic metrics (latency, burst length, packet rate, synchronization gap) | Lacks real-world large-scale deployment validation | Attack effectiveness drops significantly with loose synchronization | Up to 85.7% reduction in attack effectiveness under ~90 ms synchronization error |
| Cakmakci et al. 2020 [42] | E-KOAD(Enhanced Kernel Online Anomaly Detection) + Mahalanobis Distance + k-means | Network/Cloud DDoS detection | CICIDS2017 | K-means (threshold optimization) | Entropy-based flow features + statistical traffic features | High computational cost and threshold dependency | Hybrid statistical + ML approach improves anomaly detection accuracy | Efficient unsupervised detection of DDoS with improved anomaly identification |
| Virupakshar et al. 2020 [43] | DNN, Decision Tree, KNN, Naïve Bayes (ML+DL) | OpenStack-based cloud DDoS detection | Self-generated cloud dataset (LOIC simulated) | NA | Traffic based features ( protocol, bytes, count, rates) | Dataset is synthetic and limited to simulated attacks | DNN outperforms traditional ML models in cloud environment | Effective detection of DDoS with higher accuracy using DNN |
| Bouyeddu et al. 2020 [44] | CRPS-ES (Continuous Ranked Probability Score + Exponential Smoothing) | Network-based DoS/DDoS Detection | DARPA99, MAWI, ICMPv6 datasets | NA | Traffic features (SYN packets, ICMP messages, UDP flow) | Less effective for multi-scale patterns | Model improves anomaly detection using non-parametric thresholding | Real-time DDoS detection with high sensitivity and low false alarms |
| Kasim et al. 2020 [45] | AE-SVM(Deep + ML Hybrid) | Network anomaly detection (DDoS) | CICIDS2017, NSL-KDD + Virtual traffic | NA | Autoencoder-based feature learning & dimensionality reduction | Sensitive to hyperparameters and feature reduction settings | AE+SVM enhances anomaly detection capability and reduces false positive | High accuracy with fast detection and reduced computational cost |
| SaiSindhuTheja et al. 2021 [46] | OCSA + RNN (Hybrid DL + Optimization) | DoS attack detection in cloud computing | KDD Cup 99 | OCSA (CSA + OBL) | Implicit via RNN & selected features | High computational complexity due to metaheuristic + RNN | Optimal feature selection improves detection accuracy | High detection performance with reduced features |
| Kumar et al. 2021 [47] | Stacking Ensemble (DT +NB + RF + XGBoost) | IoMT(fog-cloud security) | ToN_IoT dataset | NA | Statistical + traffic features | Limited to binary classification | Ensemble improves detection rate | High detection with reduced false alarms |
| Thilagam et al. 2021 [48] | RC-NN (CNN + LSTM) + ALO Optimization | Cloud network IDS | DARPA + CSE-CIC-IDS 2018 | Removal of irrelevant features | Statistical + embedding features | High complexity & preprocessing dependency | Hybrid DL improves detection | High accuracy with low error |
| Agarwal et al. 2021 [49] | LSTM-based P-Estimation Deep Learning Model | Cloud security (FRC/EDoS attack detection) | NASA web Server logs | Quantile-based filtering | Wavelet transform + time-series sequences | Needs periodic retraining & high data dependency | Dynamic DL model improves low-rate attack detection | Very high accuracy with FPR = 0% and FNR = 0.0059% |
| Mohammad et al. 2021 [50] | ML-CCM (Machine Learning + Big Data Hybrid Model) | Cloud security & big data analytics | NA | Filtering of irrelevant & redundant features | Network traffic + system performance features | No standard dataset & high system complexity | ML + big data improves cloud threat prediction | Improves security, data management, and transmission efficiency |
| Kushwah et al. 2021 [51] | SaE-ELM-Ca (Self-adaptive Evolutionary Extreme Learning Machine with crossover adaption ) | Cloud computing (DDoS detection) | NSL-KDD, ISCX IDS 2012, UNSW-NB15, CICIDS2017 | NA | Network traffic statistical features | Higher training time due to evolutionary optimization | Adaptive ELM improves detection performance | Provide high accuracy across multiple datasets |
| Zhao et al. 2021 [52] | Protocol + Session Analysis + Improved C4.5 + Queuing Model (M/M/n/m) | Edge Computing (Multimedia Traffic Anomaly Detection) | Multimedia traffic + NSL-KDD + WSN-DS + CIC-IDS2018 | NA | Statistical traffic features(flow-based: packet size, interval, session duration, etc.) | Slightly reduced security when using lightweight/ targeted detection | Lightweight detection improves efficiency in edge devices | High accuracy with low cost and reduced packet loss |
| Shah et al. 2021 [53] | EDOS-IDM (SDN-based ICMP Detection & Mitigation Model with Statistical + Exponential Backoff) | Cloud Computing Environment (OpenStack with SDN | Real-time traffic (HPING3 + Ping, OpenStack testbed) | NA | Statistical features( packet size, IAT, flow duration, packet count) | Needs parameter tuning (thresholds like α, δ, τ); depends on SDN infrastructure | Efficient detection of both volumetric & normal ICMP attacks | Reduced resource usage, bandwidth, and cost (near-zero for large attacks) |
| Sambangi et al. 2022 [54] | SWASTHIKA (Similarity-based ML model + Feature Transformation) | Cloud & IoT network environments (DDoS detection) | IoT DoS & DDoS Dataset (IEEE Dataport / CICIDS-based) | NA | Similarity-based feature transformation (SWATHI function + clustering) | High non-linearity & generalization to unseen data is limited | Feature transformation reduces dimensionality and improves detection performance | Improved accuracy & detection rate after feature reduction |
| Geetha et al. 2022 [55] | FKPCA-GWO WDBiLSTM | Cloud environment (Intrusion Detection) | UNSW-NB15, NSL-KDD, BoT-IoT, TUIDS | NA | Fisher Kernel PCA (FKPCA) | High computational complexity & dependency on multiple parameters | Hybrid model improves intrusion detection by combining feature reduction and optimized deep learning | Achieves high accuracy, precision, sensitivity, specificity, and ROC performance |
| SaiSindhuTheja et al. 2022 [56] | MFSLnO + DBN + Bait Approach | Cloud ( SaaS-based SDN, IoT botnet detection) | N-BaIoT | NA | 115 statistical traffic features | High computational complexity & no real cloud deployment | Integrated detection and mitigation improves botnet defense | Packet loss=16% and throughput = 89% |
| Karthick et al. 2022 [57] | S2RF2S + SxB2IN2 | Cloud-based DDoS detection | NSL-KDD | S2RF2S (recursive feature selection) | LSAR + behavioral traffic features | Evaluated on small dataset & simulation setup | Feature selection + neural model improves detection | Better accuracy & reduced attack time |
| Mayuranathan et al. 2022 [58] | EOS-IDS (IHO + CRDO + DKNN) | Cloud intrusion detection | DARPA IDS, CSE-CIC-IDS2018 | CRDO(Red Deer Optimization) | Traffic statistical features (CICFlowMeter) | High complexity due to hybrid model | Hybrid DL improves IDS performance | High detection accuracy & low error |
| Alam et al. 2022 [59] | SVM-DEHO | Cloud DDoS detection | NSL-KDD, UNSW-NB15, ISCXID, CIC-IDS2017 | KPCA | Traffic flow features (preprocessed) | High computational & training time due to hybrid optimization | Optimization improves SVM performance | High detection performance outperforms V-ELM, DBN, SaE-ELM, FCM-SVM |
| Khoda Parsat et al. 2022 [60] | Survey-based security framework | Cloud security (DoS/DDoS, authentication, vulnerabilities) | NA | NA | NA | No experimental validation | Reviews multiple security mechanisms | Provides comprehensive cloud defense strategies |
| Abdullayeva et al. 2022 [61] | PCA + DBSCAN, PCA + Agglomerative, PCA + K-means | DDoS detection in E-government cloud | CSE-CIC-IDS2018, NSL-KDD, HTTP CSIC 2010 | PCA | PCA( Dimensionality Reduction) | Sensitive to parameter tuning & clustering limitations | Features reduction improves clustering performance | PCA+DBSCAN achieved best ARI = 0.8989 |
| Lata et al. 2022 [62] | Comparative Survey of IDS( Signature-based, Anomaly-based, VMI, HVI, Hybrid) | Cloud Computing Security (Intrusion detection System in Cloud) | KDD99, NSL-KDD, UNSW-NB15, CICIDS2017, CSE-CIC-IDS2018 | NA | NA | Existing IDS techniques lack a unified model and rely on outdated or incomplete datsets. | Hybrid IDS combined with feature selection significantly enhances detection accuracy and reduces false alarm rates | Highlighting hybrid approaches as the most effective for improving detection performance |
| Shah et al. 2022 [63] | EDOS-TSM (Statistical anomaly detection with binomial distribution) | Cloud computing security (EDOS attack mitigation using SDN) | Real-time OpenStack traffic (HPING3 for attack, D-ITG for normal) | Threshold-based filtering using flow statistics and probability | TTL value, TCP SYN flags, flow statistics (packets, flows), IP addresses, ports | Does not consider SDN controller cost, computational overhead, and scalability | EDOS-TSM accurately detects TCP SYN-based EDOS attacks using probability and TTL analysis | Achieves low false negatives and efficient resource utilization compared to existing methods |
| Sharma et al. 2022 [64] | Decision Tree, K*, Randomizable Filter + Ensemble Voting (AI-Ml based risk model) | Cloud computing security(risk assessment & threat analysis) | Synthetic dataset (1940 instances, 18 risk factor) | Best first, Random Search, Ranker (via WEKA/Orange) | Risk factors (A&AC,SLA,DB,TPM,etc.) encoded into numerical values | Synthetic dataset; lacks real-world validation; limited scalability analysis | Ml-based risk assessment improves prediction of cloud security risks | Decision Tree & K* provide best performance |
| Kamal et al. 2022 [65] | Genetic algorithm + Selective Encryption + Parallel Distribution (Hybrid) | Cloud security (distributed cloud storage & data confidentiality) | Synthetic datasets (Data pools: 1KB-500MB from UCI, GitHub, etc.) | Classification-based (Fuzzy, Logistic Regression, SVM-optional pre-processing) | Data splitting (R, C) + GA crossover + XOR-based encoding (IDED) | Limited to text data; lacks heterogeneous data validation | GA-based framework improves confidentiality with optimized time & memory | Outperforms AES & ICA in efficiency, throughput, and memory usage |
| Ismail et al. 2022 [66] | Random Forest, XGBoost | DDoS attack classification & prediction | UNSW-NB15 dataset (GitHub source) | NA | Label encoding + statistical preprocessing | Limited feature engineering; no advance selection techniques; dataset specific validation | Random Forest achieved balanced precision & recall, XGBoost slightly higher | Improved accuracy compared to existing works, showing effectiveness of ensemble ML models |
| Agarwal et al. 2022 [67] | FS-WOA-DNN (Feature Selection + Whale Optimization + Deep Neural Network) | DDoS attack detection in cloud storage environment | CICIDS2017 | Whale Optimization Algorithm (FS-WOA) | Min-Max normalization + optimized feature subset selection | Higher computational complexity (optimization + DNN); requires proper feature tuning; depends on dataset quality | Optimal feature selection significantly improves detection accuracy and reduces false rates | Achieved 95.35% accuracy, high sensitivity (90.71%) and specificity (96.9%), outperforming SVM, KNN, ANN, and DNN |
| Chundru et al. 2022 [68] | Decision Tree (DT) Classifier | DDoS intrusion detection in cloud networks | BoT-IoT dataset (UNSW Canberra Cyber Range) | NA | One-hot encoding feature mapping, normalization, correlation analysis | Dataset imbalance (more attack samples than normal); limited comparison with advanced DL models | Decision Tree effectively captures DDoS patterns with high interpretability and efficiency | Outperforms Gradient Descent and Random Forest |
| Esmaeili et al. 2022 [69] | BiLSTM, LSTM, MLP, KNN, SVM, LDA, DT, RF | DDoS attack detection in IoT environment | NSl-KDD dataset | NA | Flow-based and packet-based feature extraction normalization | Dependence on NSL-KDD dataset limitations; overfitting observed between training and testing | Sequential deep learning models ( especially BiLSTM ) are more effective in capturing complex attack patterns compared to traditional Ml models | BiLSTM provides the most reliable and consistent detection performance with superior generalization |
| Priyadarshini et al. 2022 [70] | LSTM-based Deep Learning +SDN | DDoS mitigation in fog & cloud environment | ISCX IDS + Botnet dataset (combined) | NA | Traffic feature extraction with encoding (binary & categorical) | Complex architecture (SDN + DL); requires continuous training and tuning | LSTM effectively learns temporal traffic behavior and identifies malicious packets | Model provides reliable detection and successfully filters malicious traffic while allowing legitimate data in fog-based systems |
| Gaber et al. 2023 [71] | KPCA + CNN (hybrid DL) | Metaverse-IoT intrusion detection | BoT-IoT, ToN-IoT | KPCA | KPCA dimensionality reduction | Tested only on benchmark datasets | Hybrid KPCA-CNN improves attack detection in metaverse IoT | High detection capability across multiple IoT attacks |
| Kotel et al. 2023 [72] | Blockchain-based Hyperledger Fabric architecture | Smart home IoT security | No public dataset (architecture-based evaluation) | NA | NA | No experimental validation on real datasets | Blockchain improves access control and secure IoT communication | Enhanced security, privacy, and resilience against attacks |
| Yuan et al. 2023 [73] | AMLFN-AD (Adaptive Multi-Level Fusion Ensemble) | Intelligent building intrusion detection | BoT-IoT, CICIDS2018, NSL-KDD | Hybrid model selection based on accuracy & correlation | Data preprocessing (SMOTE + undersampling + MinMax normalization + one-hot encoding) | High computational cost due to multiple model training | Multi-level adaptive ensemble improves attack detection | Achieved superior performance over MTH-IDS, CNN-BiLSTM and XGBoost |
| Yang et al. 2023 [74] | Cloud-Edge Coordinated IOSARIMA + CNN | Industrial Cyber-Physical Systems (ICPS) | Real ICPS testbed (power generation, transmission, distribution) | Feature reduction using IP-based aggregation | CICFlowmeter + feature reduction + grayscale transformation | Complex architecture; requires edge-cloud infrastructure | Reduces traffic load and improves detection accuracy | Achieves higher accuracy and lower FPR |
| Mustapha et al. 2023 [75] | GAN-based adversarial attack framework | DDoS intrusion detection systems | Not dataset specific (general IDS evaluation) | NA | Feature perturbation using GAN-generated samples | Focuses on adversarial evaluation not detection model | GAN-generated adversarial samples reduces IDS detection stability | Shows vulnerability of ML-based IDS to adversarial attacks |
| Lai et al. 2023 [76] | MIC-GA + Advanced online MPA optimized IDS | DoS Detection in WSN | WSN traffic dataset | MIC + Genetic Algorithm | Dimensionality reduction preprocessing | Optimization increases computational cost | Optimal feature subset improves detection | Enhanced DoS detection |
| Song et al. 2023 [77] | Anomaly detection + CUSUM + CART + MLP( Hybrid) | Public cloud intrusion detection | Public Cloud IDS datasets (A, B, C) | mRMR, Information Gain, Pearson correlation, Mutual Information | Feature matrix construction | Complex architecture | Feature selection improves accuracy | Improved intrusion detection |
| Zhou et al. 2023 [78] | CoWatch + LSTM | SDN-based Edge Computing DDoS detection | CIC- DDoS2019, CIC-IDS2017, UNSW-NB15, CSE-CIC-IDS2018, ISCX-IDS2012 | Optimal threshold filtering | Flow-based traffic features | Communication overhead in distributed SDN | Collaborative LSTM improves prediction | High detection accuracy |
| Pasha et al. 2023 [79] | LRDADF + (SAE+ CNN) with HA-LRDD | Low-rate DDoS detection in cloud | CIC-DDoS2019 | NA | Statistical + SAE-based feature learning | High computational complexity (deep model) | Hybrid SAE-CNN improves feature learning and detection | High detection rate with low FPR |
| Hezavehi et al. 2023 [80] | IDARP (Anomaly-based framework) | Cloud Service Providers (Public & private CSP) | Simulated cloud traffic | NA | Traffic behavior monitoring | Higher false alarms in small CSP | Interactive filtering improves mitigation | Acceptable detection accuracy |
| Selvan G.S.R. et al. 2023 [81] | FACVO-based DNFN (Deep Neuro-Fuzzy Network) | Cloud Computing | NSL-KDD, BoT-IoT | NA | RV coefficient + Deep QNN feature fusion | Not suitable for real-time applications | Feature fusion with FACVO improves detection | Accuracy above 92% |
| Abdullayeva 2023 [82] | Cyber Security & Cyber Resilience Reference Model | Intelligent cloud computing systems | NA | NA | NA | No experimental validation | Integrated security + resilience improves cloud protection | Conceptual model for secure cloud architecture |
| Farhat et al. 2023 [83] | XGBoost-based Ml IDS (NB, LR, RF comparison) | Cloud Environment | CIC-IDS2017 | Extra Tree feature | Z-score normalization | Tested on single dataset only | XGBoost outperforms other Ml models | Accuracy 99.11%, FAR 0.011% |
| Islam et al. 2023 [84] | Ensemble (SVM + KNN + LR) | Cloud DoS/ DDoS detection | IIoT Cloud Dataset (Kaggle) | Data Cleaning & balancing | Network flow statistical features | Memory overload affects performance | Ensemble improves detection accuracy | Improved detection performance |
| Ali et al. 2023 [85] | SVM, KNN, DT, MLP, CNN | SDN-based DDoS detection | CICIDS2017, CICDDoS2019 | Feature reduction (remove irrelevant features) | Flow-based statistical features | Limited dataset (one-day) & offline evaluation | SVM provides stable and efficient detection | SVM achieved better prediction accuracy |
| Ali et al. 2023 [86] | ML/DL survey | DDoS detection in IoT, SDN, network environments | KDD Cup99, Kyoto 2006+, NSL-KDD, UNSW-NB15, CIC-IDS2017, CSE-CIC-IDS2018, ISCX2012, CICDDoS2019 | NA | NA | Lack of lightweight real-time models & dataset diversity | ML/DL improve DDoS detection accuracy | High accuracy but needs real-world validation |
| Sadhwani et al. 2023 [87] | ML-based lightweight IDS (LR,NB,RF,ANN,KNN) | IoT-based DDoS detection | TON-IoT, BoT-IoT | ExtraTreeClassifier | Data standardization + label encoding | Limited to IoT datasets and offline testing | Lightweight feature-reduced model improves detection | Efficient DDoS detection with reduced computation |
| Deniz et al. 2023 [88] | ANN, CNN, LSTM (DL Models) | Cloud-based DDoS detection | OpenStack simulated cloud dataset (HTTP Flood) | NA | Scapy-based packet feature extraction from PCAP | Limited dataset and simulated environment | LSTM performs better for cloud DDoS detection | Deep Learning improves cloud DDoS detection performance |
| Balasubramaniam et al. 2023 [89] | GHLBO(Gradient+HLBO)-optimized DSA (Deep Learning) | Cloud computing DDoS detection | NSL-KDD, BoT-IoT | GHLBO optimization | Feature fusion + data augmentation | High computational complexity | Optimization improves detection performance | Accuracy = 91.7%, TPR= 0.908, TNR= 0.909 |
| Ahmed Issa et al. 2023 [90] | Hybrid CNN-LSTM | Network DDoS detection | NSL-KDD | NA | CNN automatic extraction + LSTM temporal learning | Computationally intensive architecture | Hybrid deep learning improves detection | Proposed model outperforms individual CNN and LSTM |
| Najar et al 2024 [91] | BRS + CNN | SDN-based DDoS detection | CICDDoS2019 | Balanced Random Sampling + Info Gain | CNN automatic feature extraction | Requires large training dataset and additional features for similar attack types | Integrated CNN with sampling improves detection and mitigation | High-performance SDN-based DDoS detection with notification and mitigation support |
| Abdullayeva et al. 2024 [92] | GraphSAGE & GCN (GNN) | Cloud-based DoS/DDoS detection | CSE-CIC-IDS2018 | NA | Graph topology-based traffic feature vectors | Requires graph construction and simulation dataset | GraphSAGE performs better than GCN for cloud attack detection | Graph-based learning improves detection using node-edge relationships |
| Pandithurai et al. 2024 [93] | HBO + Bi-LSTM | Cloud DDoS detection | CICIDS2017, CICIDS2018, CIC-DoS2016, Kaggle dataset | Honey Badger Optimization (MSE-based) | Bi-LSTM temporal learning | Multi-stage pipeline increases computational complexity | Optimization improves feature quality for DDoS detection | Bi-LSTM optimized features enhances detection performances |
| Ouhssini et al. 2024 [94] | DeepDefend (CNN-LSTM-Transformer + AutoCNN-DT) | Cloud DDoS detection | CIDDS-001 | Genetic Algorithm | Entropy forecasting + CNN autoencoder | Complex multi-stage architecture | Entropy-based forecasting improves anomaly detection | Framework enables real-time detection and prevention |
| Farzaneh et al. 2024 [95] | Deep Transfer Learning (BiLSTM, CNN, ResNet, Inception) | DDoS detection in 5G network slicing | 5G dataset + 5G-NIDD | PCA | CICFlowMeter (84-> 8 features) | High training time and dependency on labelled source data | Transfer learning significantly improves detection under limited data | BiLSTM achieved highest performance with strong accuracy, recall, precision, and F1-score |
| Jayakumari et al. 2024 [96] | Cloud-based Hybrid Blockchain E-Voting Framework | Secure cloud e-voting system | Simulated voting network data | NA | Timestamp authentication + PBFT consensus | Limited real-world deployment validation | Hybrid blockchain improves security and transparency of voting | System enhances reliability and reduces delay in voting operations |
| Alani 2024 [97] | SDN-based ML Edge detection with RFE | Smart city IoT security | InSDN dataset | Recursive Feature Elimination | Packet-level traffic features | Requires preprocessing and dataset balancing | Feature reduction improves efficiency without degrading detection performance | System enables packet-level attack detection in SDN edge environments |
| Binbusayyis 2024 [98] | RBF-SVR + VGG19-2D CNN | Network intrusion detection/ DDoS cloud-fog environment | UNSW-NB15, CICIDS2017, CICIDS2018 | Wrapper based FS using RBF-SVR | CNN feature learning via VGG19 | High computational requirement due to deep CNN architecture | Feature selection reduces dimensionality and improves classification performance | Hybrid deep learning model improves intrusion detection capability with reduced false alarms |
| Shang 2024 [99] | Gaussian naïve Bayes (with Random Forest comparison) | DDoS detection in cloud environment | Wireshark generated packet dataset | Normalization using WEKA | Statistical probability features (mean & deviation) | Not applicable for real-time monitoring | Naïve Bayes effectively identifies malicious packets | Improves prediction of DDoS traffic |
| Kumar et al. 2024 [100] | Two-Layer LDS + Load Balancer + Container-based mitigation | Cloud-based DDoS mitigation | Simulated container-based cloud traffic | Threshold-based filtering | Traffic aggregation & bandwidth control | Threshold. Dependency may misclassify traffic | Integrating resource reservation and bandwidth limiting improves mitigation | Reduces response time and preserves benign services |
| Rahman et al. 2024 [101] | Linear vs Nonlinear ML classifiers (LR, LDA, NB, DT, RF, GBM, KNN, XGB) | Machine Learning based Intrusion Detection | WUSTL-EHMS-2020, UNSW-NB15 | Preprocessing & statistical validation used | Statistical feature analysis with nonlinear decision boundaries | Higher computational complexity due to multiple classifiers and statistical validation | Nonlinear classifiers capture complex intrusion patterns better than linear models | Nonlinear classifiers significantly improved intrusion detection accuracy, generalization capability, and robustness compared to linear classifiers |
| Asha S. et al. 2024 [102] | Taxonomy based Survey | Insider Threat in Cloud | Multiple benchmark datasets (surveyed) | NA | NA | No model implementation | Provides structured taxonomy of insider threats and defensive mechanisms | Useful classification framework for future insider threat research |
| Abdullah et al. 2024 [103] | ML & DL survey | Cloud Anomaly Detection | Multiple dataset reviewed | NA | NA | Lack of real-world validation and scalability issues | Identifies research gaps in IDS and DDoS detection for cloud networks | Highlights Need for scalable and adaptive IDS solutions |
| Afraji et al. 2024 [104] | Deep learning based DDoS defense survey | Cloud Cybersecurity | Multiple datasets reviewed | NA | NA | Dataset imbalance and high resource requirement | Deep learning improves adaptive detection | Supports explainable and scalable DDoS defense |
| Shaikh et al. 2024 [105] | CNN + Vision Transformer (ViT) hybrid model | Cybersecurity/ DDoS detection | CICDDoS2019 | PCA + SMOTE | CNN feature extraction + Transformer attention | High computational complexity and dependency on preprocessing | ViT outperforms CNN in detecting complex attack patterns | Hybrid model improves detection accuracy and efficiency |
| Qureshi et al. 2025 [106] | Hybrid LSTM-GRU IDS | Cloud-based IIoT intrusion detection | Kitsune-2019 | MinMax normalization | LSTM + GRU hybrid deep feature learning | High computational cost and dependency on dataset preprocessing | Hybrid model captures long-term and short-term dependencies effectively | Improved detection accuracy with reduced training time |
| Sharma et al. 2025 [107] | Word2Vec + LSTM + Softmax | Cloud service attack classification (IaaS, PaaS, SaaS) | Cloud threat text corpus (CSA, AWS, Microsoft, IBM, CrowdStrike blogs) | CBOW word representation | Word2Vec embeddings + LSTM sequential learning | Overlapping attack descriptions reduce model discrimination | LSTM effectively captures contextual relationships in attack descriptions | LSTM provided best classification performance among models |
| Goyal et al. 2025 [108] | ICO + Dual Attention Dense Bi-GRU | Malicious behaviour detection in cloud computing | Microsoft Malware, GitHub host list, CIC-IDS2017, CIC-IDS2018, NSL-KDD+ | Improved Cheetah Optimization (ICO) | Dual Attention + DenseNet + Bi-GRU | Requires High computational resources for deep attention-based architecture | Dual attention with Bi-GRU improves malicious behaviour detection accuracy | Proposed model achieved superior detection performance across multiple datasets |
| Wang et al. 2025 [109] | RAD (Runtime Reliability Anomaly Detection) | DDoS reliability monitoring in cloud-based SaaS systems | serviceSurvey, WS-DREAM (public cloud API) | NA | SVD + Jensen-Shannon divergence + FDC | Requires runtime parameter tuning and may be sensitive to reliability fluctuations | Concept-drift-based reliability analysis improves DDoS anomaly detection | RAD improves runtime reliability detection in cloud-based APIs |
| Selvam P et al. 2025 [110] | SVCI-TIPNN-AD | Cloud intrusion detection in virtualized infrastructure | CSE-CIC-IDS2018, CICDDoS2019 | BBOA optimization | EMWT (Holoentropy + EMA features) | Depends on preprocessing and balanced data | Optimized TIPNN improves temporal attack detection | Higher detection accuracy with reduced complexity |
| Abiramasundari et al. 2025 [111] | PCA-based EDAD (RF, SVM, KNN, LR, DT) | DDoS detection in cybersecurity networks | CICIDS2017, CICIDS2018, CICDDoS2019 | PCA | Dimensionality reduction (variance-based) | Lacks real-time deployment and deep learning comparison | PCA + ML improves detection accuracy and reduces dimensionality | RF achieved highest accuracy |
| Mandela et al. 2025 [112] | BERT, DNN, RNN, LSTM, Bi-LSTM, GRU | Network-based DDoS detection | CIC-DDoS2019 | Correlation based feature reduction | Composite feature engineering + temporal windowing | Transformer models computationally heavy and less suitable for structured data | Sequence models capture temporal pattern better | RNN achieved highest performance |
| Bahashwan et al. 2025 [113] | Deep learning-based MLP | DDoS detection in SDN | SDN DDoS attack dataset | SMOTE + normalization + label encoding | Multi-layer representation learning( MLP hidden layers) | Hyperparameter tuning required for optimal performance | MLP effectively captures complex SDN traffic patterns | MLP improves detection accuracy and reduces false positives |
| Mehmood et al. 2025 [114] | Hybrid CNN-MLP + Bayesian Optimization | SDN-based DDoS detection | CICDDoS-2019, InSDN | SHAP (Shapley Additive Explanations) | CNN spatial features + MLP deep feature learning | High Computational cost and complexity | Hybrid model improves SDN traffic patterns learning | High accuracy and reduced false positives |
| Bellmunt et al. (2026) [115] | Federated Transfer Learning (FTL) + CNN | Intrusion detection in heterogeneous 5G/IoT networks | BoT-IoT, UNSW-NB15 | Common traffic features were selected for cross-dataset transfer | CNN-based automatic feature learning; pre-trained CNN with fine-tuning of selected layers for target-domain data | Communication and computational overhead in federated training; performance is affected by heterogeneous and imbalanced local data distributions | FTL improved detection of unknown attacks and was more than conventional Federated Learning, particularly for nodes with limited malicious samples | 91.2% Accuracy for the most imbalanced node vs. 74.1% with FL; unknown-attack detection improved from 19.09% to 81.704% |
| Sun et al. (2026) [116] | LogMeta: MAML + RoBERTa + Attention-based Bi-LSTM | Few-shot, robust and adaptive log anomaly detection across heterogeneous software systems | BGL, HDFS, Thunderbird, Spirit | Cosine-similarity-based selection (CSS) to select representative log sequences | RoBERTa embeddings + Attention-based Bi-LSTM for semantic and sequential feature extraction | Limited labeled training data and heterogeneous log formats remain challenging | Integrating MAML with the hybrid RoBERTa-Attention-Bi-LSTM architecture improves robustness, rapid adaption, and cross-dataset generalization under limited labeled data | F1= 0.971 on BGL; LogMeta achieved 2-3% higher F1 than non-MAML variants on BGL and Thunderbird, with stronger gains under small-data settings |
| Zhang et al. (2026)  [117] | MAML- SAMIoT (MAML + Supervised Contrastive Loss + ALFA + MSL) | Few-shot IoT intrusion detection in cloud-fog-edge environments | FSIDS-IoT and FSIDS-IoT-V1 | Not explicitly specified | CNN-based feature learning with supervised contrastive representation learning | Limited attack samples, heterogeneous IoT environments, and communication/ computational constraints | MAML-SAMIoT improves adaptation and generalization for few-shot and unknown-attack detection | 5-way 1-shot: 73.32% Accuracy, 71.18% F1; 5-way 5-shot: 80.40% Accuracy, 80.45% F1; 5-way 10-shot: 82.65% Accuracy, 82.60% F1 |
| Ju et al. (2026) [118] | TayMAML (MAML-based Meta-Reinforcement Learning) | Dynamic heterogeneous edge computing task scheduling | DAG-generated task graphs, Alibaba Cluster Data V2017 | Not specified | Task, edge-node, terminal-device configuration and channel-state features | Assumes relatively stable task distributions and bounded heterogeneity; limited adaptability to out-of-distribution tasks | TayMAML improves generalisation and stability while reducing scheduling overhead in heterogenous edge environments | Achieved the highest generalisation and stability among compared MRL methods, with the lowest cumulative weighted cost |
| Ijaz et al. (2026)  [119] | MAML; Prototypical Network; FC-Nst baseline | Few-shot and multiclass zero-day intrusion detection under data scarcity | CICIDS2017, CICIoT2023, CIC-UNSW-NB15 | Constant-feature removal and perfect-correlation (r=1) based feature reduction | Learned embeddings through Prototypical Network | MAML has higher computational cost and longer training/inference time; performance can decline under difficult distribution shifts and unseen attacks | Meta-learning effectively adapts to previously unseen attack categories with only a few labeled samples | MAML achieved 96.67% accuracy/ 97.54% recall on CICIDS2017, 92.87% / 92.99% on CICIoT2023, and 83.20% / 83.50% on CIC-UNSW-NB15 |
| Chen & Ma (2026) [120] | Improved CNN + Model-Agnostic Meta-Learning (MAML) | Few-shot network intrusion/attack detection with emphasis on unknown and evolving attacks | Custom dataset constructed from multiple network-attack data pools; simplified R-S- Dataset contains 43,681 samples and 13 DDoS attack types | XGBoost-based top 20 features+ PCA | CNN-based 2D feature representation | High computational cost and hyperparameter sensitivity | Few-shot CNN-MAML improved adaption to unknown attacks with less labeled data | 95.31% accuracy, 0.97 AUC, 1.18% FPR |
